# Supplementary material for: Cholesterol Interaction Sites on the Transmembrane Domain of the Hedgehog Signal Transducer and Class F G Protein-Coupled Receptor Smoothened
Source: Structure. 2019 Mar 5;27(3):549–559.e2. doi: 10.1016/j.str.2018.11.003 (PMC6408332; doi:10.1016/j.str.2018.11.003)
Supplement: Document S1. Figures S1–S7 and Table S1 [file mmc1.pdf]

Structure, Volume *27*

Supplemental Information

Cholesterol Interaction Sites on the Transmembrane Domain of the Hedgehog Signal Transducer and Class F G Protein-Coupled Receptor Smoothened

George Hedger, Heidi Koldsø, Matthieu Chavent, Christian Siebold, Rajat Rohatgi, and Mark S.P. Sansom

Supplemental Information

Cholesterol and PIP_2 interaction sites on the transmembrane domain of the hedgehog signal transducer and Class F GPCR Smoothened

George Hedger, Heidi Koldsø, Matthieu Chavent, Christian Siebold, Rajat Rohatgi, and Mark S. P. Sansom

Table S1. Comparison of lateral lipid-protein binding energies for integral membrane proteins extracted from CG PMF calculations *via* umbrella sampling. Related to Figure 6.

Protein	Lipid	Well-depth (kJ/mol)	Membrane composition
Cytochrome C Oxidase[a] (Arnarez et al., 2013; Arnarez et al., 2016)	Cardiolipin^{-2}	-32 (Site I, II), -9 (Site III)	PC (100%), also tested in PC(50%):PE(50%) for Site II yielding no significant deviation.
	Cardiolipin^{0}	-21 (Site II)	PC (100%)
	PG^{-1}, PG^{-2}	-19, -24 (Site II)	
	PC	-10 (Site II)	PC (100%)
	TGL	< RT (Site II)	PC (100%)
ADP/ATP Translocase (Hedger et al., 2016b)	Cardiolipin^{-2}	-22, -20, -24 (Site I, II, III)	PC (100%)
	PS	-17 (Site I)	PC (100%)
	PC	< RT	PC (100%)
Epidermal Growth Factor Receptor Transmembrane Dimer (Hedger et al., 2016a)	PIP$_2^{-5}$, PIP$_2^{-3}$, PIP$_2^{c}$	-42, -35, -13	PC (100%)
	PS	-9	PC (100%)
	GM3, GM3 (-Neu5Ac)	-9, -4	PC (100%)
	PG	-5	PC (100%)
	PC	< RT	PC (100%)
Kir2.2[b] (Domański et al., 2017)	PIP$_2^{-5}$	-45	PC (100%)
P-glycoprotein (Domicevica et al., 2018)	Cholesterol	-25, -6, -4 (Sites I, II, III)	PC (100%)
Aquaporin-1[d] (Gu et al., 2017)	GM3	-24 to -19	PC (100%)

[a]These studies include a number of other calculations for various tail saturation patterns and cardiolipin derivatives. Only the major lipid types are catalogued here. See the original references for further details (Arnarez et al., 2013; Arnarez et al., 2016).[b]Calculations for this system were performed using replica exchange umbrella sampling. [c,d]GM3 profiles for interaction with Aquaporin-1 were performed using a several water models, only the range of values obtained is given here. See the original paper (Gu et al., 2017) for further details.

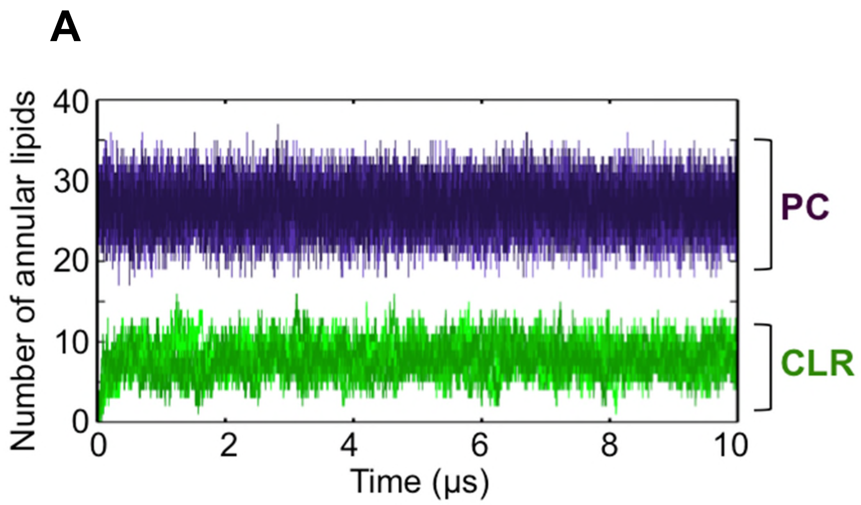

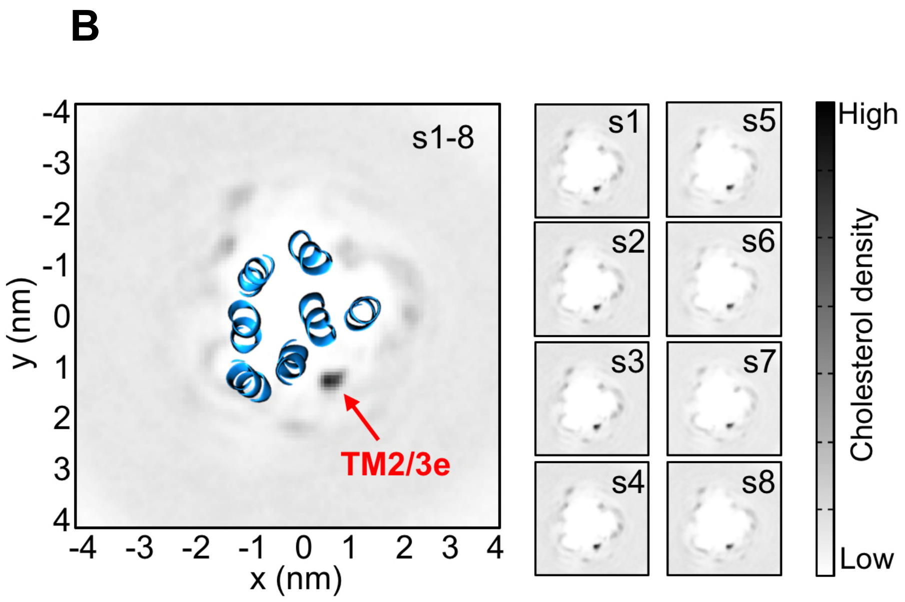

Figure S1: Convergence tests. Related to Figure 2.

A. Annular lipid composition. Number of PC and cholesterol (CLR) annular are shown as a function of simulation time. Data is shown for 8 x 10 μs of CGMD. Annular lipids are defined as those within an 6 Å cutoff distance from the protein. **B.** Convergence of independent CG-MD simulations. 2D time-averaged density projections for membrane cholesterol around SMO. Left: Density averaged over 8 independent simulations each of 10 μs duration of CGMD, Right: Density projections from each individual repeat simulation. Each repeat simulation (s1-8) was initiated from a different random distribution of lipids, waters, and ions around the protein. Invariably all simulations converged on the same density pattern, pointing to TM2/3e as a putative cholesterol interaction site.

A

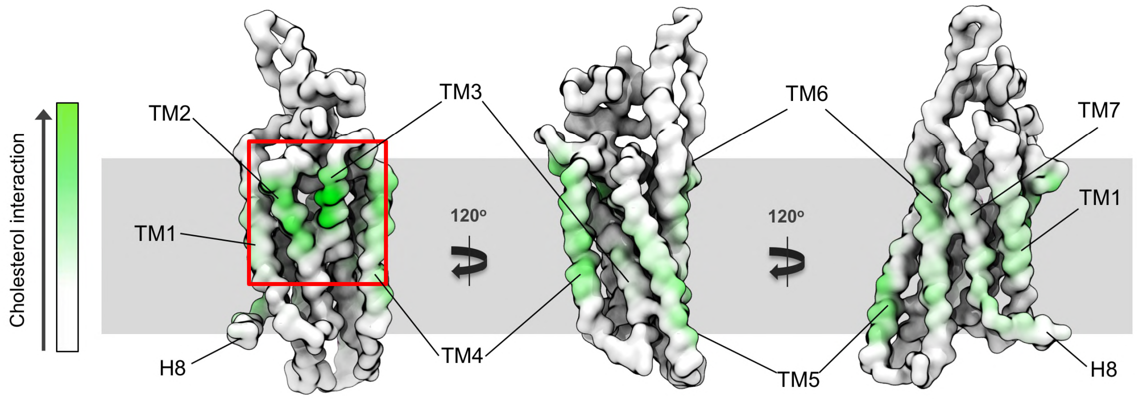

B

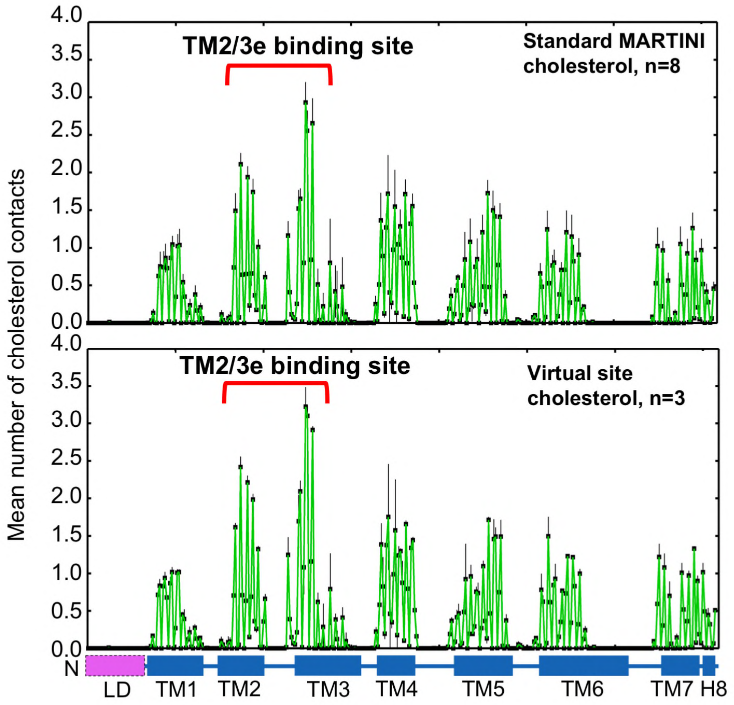

Figure S2. Cholesterol contacts. Related to Figure 3.

A. Global view of cholesterol contacts mapped onto structure. Each residue is colored according to the mean number of cholesterol contacts formed per frame of the simulation ("cholesterol interaction"), from white (no contact) to green (high contact) using contacts extracted from 8 x 10 µs of CGMD. The TM2/3e binding site is delineated by a red box. Contact analysis was performed using a 6 Å distance cutoff to define 'contact', based on the radial distribution function for CG Martini lipid-protein interactions. Further details of the contact analysis are described in the Methods section. **B.** Comparing cholesterol parameters. Cholesterol contact maps for (upper panel) the standard cholesterol model (Marrink et al., 2008), and (lower panel) the virtual site cholesterol model (Melo et al., 2015). *n* denotes the number of simulation repeats. Black error bars denote the standard deviation.

A

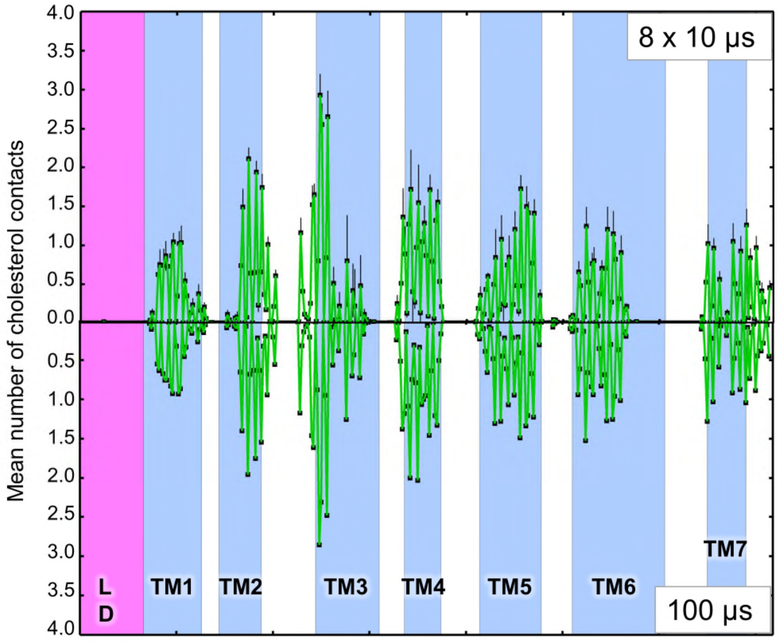

B

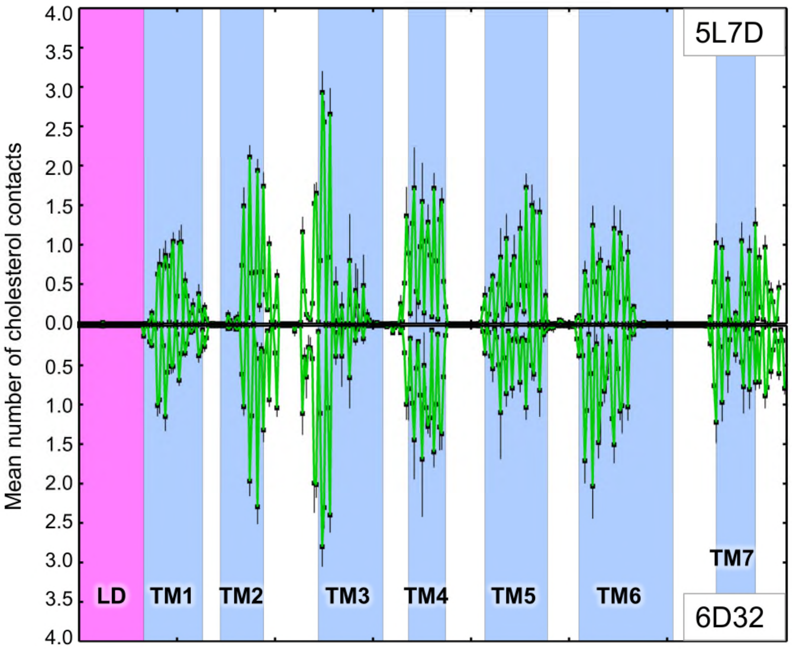

Figure S3. Cholesterol contacts. Related to Figure 3.

A. Time dependent convergence of cholesterol contacts within equilibrium CGMD simulations. Cholesterol contacts are shown for an ensemble of 8 x 10 µs of CGMD (top), and for a single 100 µs CGMD simulation (bottom). Black error bars denote the standard deviation (*n*=8). The contact maps are identical within the errors derived from the smaller ensemble. **B.** Conformational dependence of cholesterol contacts. Cholesterol contacts are shown for a structure of the human 7TMD (PDB id: 5L7D) (top), and for a recently emerged alternative conformational from *X. laevis* (PDB id: 6D32) (bottom). In both cases contacts were extracted from an ensemble of 8 x 10 µs of CGMD. Black error bars denote the standard deviation (*n*=8). The high levels of contact around TM2/3e are preserved in both structures.

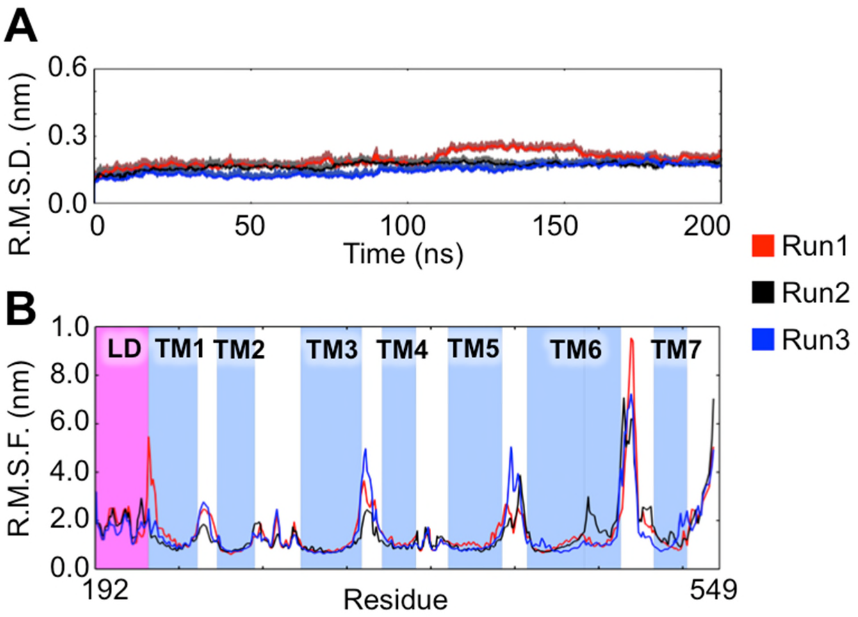

Figure S4. SMO stability within atomistic simulations. Related to Figure 5.

A. Root mean square deviation (RMSD) for Cα particles within helical secondary structure, calculated after fitting to the crystal structure coordinates (Byrne et al., 2016). The values reach a stable plateau of ca. 0.2 nm for all three repeats simulations. **B.** The predominant structural flexibility was seen in disordered loop regions, the C-terminal tail, and particularly ECL3 connecting TM6 and TM7. These observations are in agreement with our previous all-atom simulations of the full-length construct containing the CRD (Byrne et al., 2016).

A

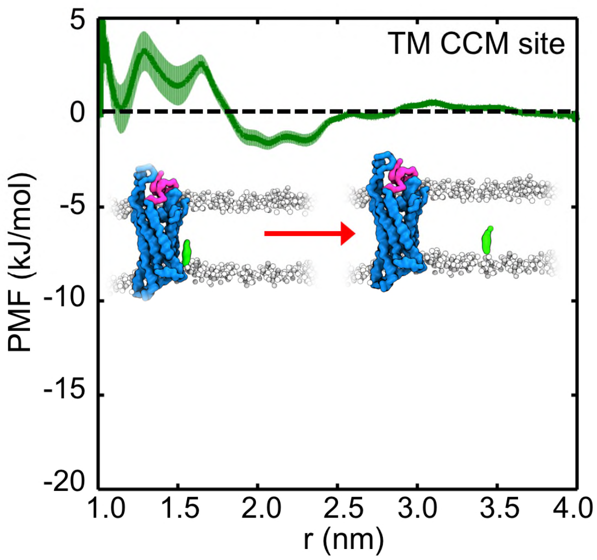

B

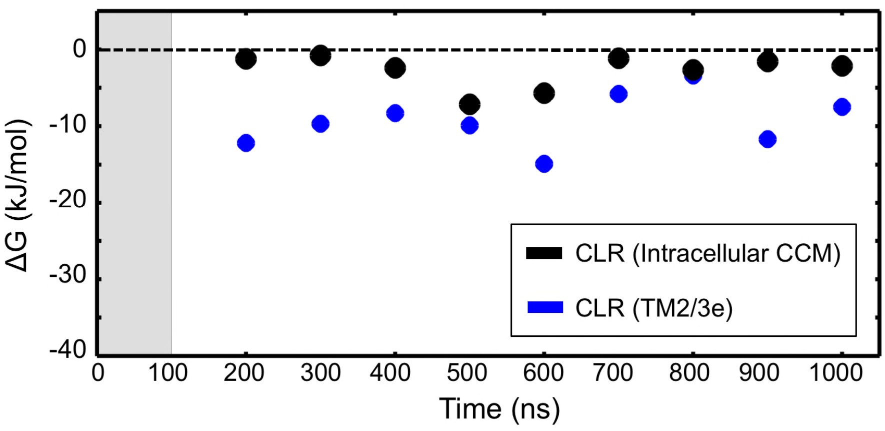

Figure S5. PMF analysis. Related to Figure 6.

A. PMF for cholesterol interaction with cholesterol consensus motif (CCM) (Hanson et al., 2008). Insets depict simulation snapshots of bound and unbound cholesterol. The shaded areas behind each curve indicate the standard deviation estimated from bootstrapping. The well-depth is < 2.5 kJ/mol (RT), indicating no significant interaction at this region, in agreement with the blind predictions of the equilibrium simulations. **B.** Well-depths calculated for multiple, non-overlapping 100 ns segments of CGMD are shown as a function of time. The first 100 ns of each window were excluded as equilibration. The patterns obtained here may be compared to those of Cardiolipin (also shown here) binding to the ADP/ATP Translocase we previously reported (Hedger et al., 2016b).

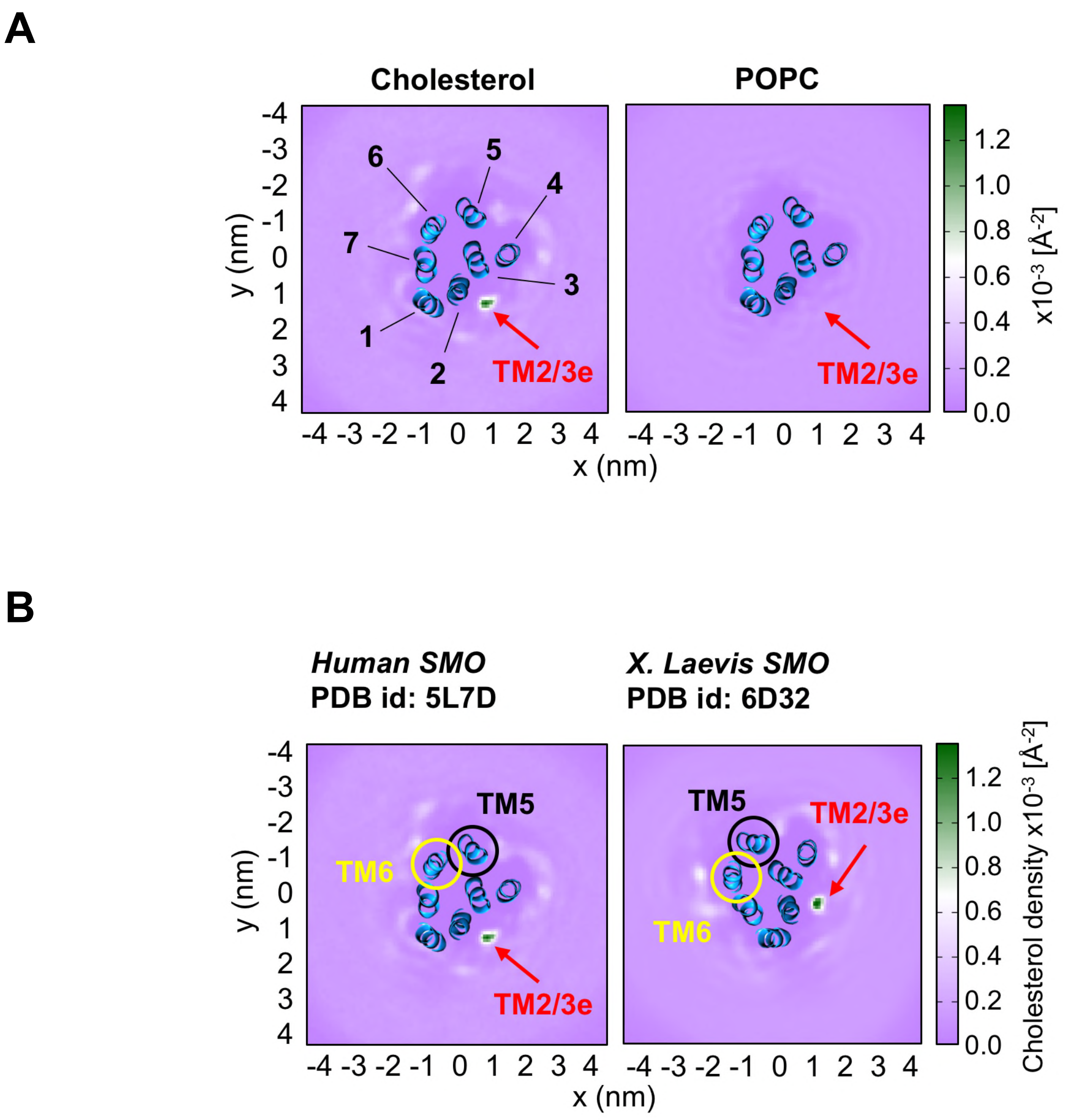

Figure S6: Cholesterol density maps. Related to Figure 2.

A. (left panel) Cholesterol density around SMO computed from 8 x 10 μs of CGMD. (right panel) POPC density around SMO in a set of control CGMD simulations totaling 3 x 10 μs in a POPC bilayer containing no cholesterol. In the absence of cholesterol POPC showed no specific interaction sites, instead forming a characteristic anulus around the protein and interacting in a non-specific fashion. See Methods for details of how density calculations were performed. **B.** Dependence of cholesterol density maps on structure. 2D cholesterol density maps are shown for CGMD simulations using the structure of human SMO (left) and the recently determined alternative conformation of the *X. laevis* structure (right). To aid comparison TM5 is encircled in black and TM6 in yellow. The location of TM2/3e is indicated by a red arrow. The primary cholesterol binding site in both sets of simulations is TM2/3e, with only a low level of diffuse density observed around TM5 and TM6. See Table 1 for a summary of the simulations, and Methods for a description of how the density calculations were performed.

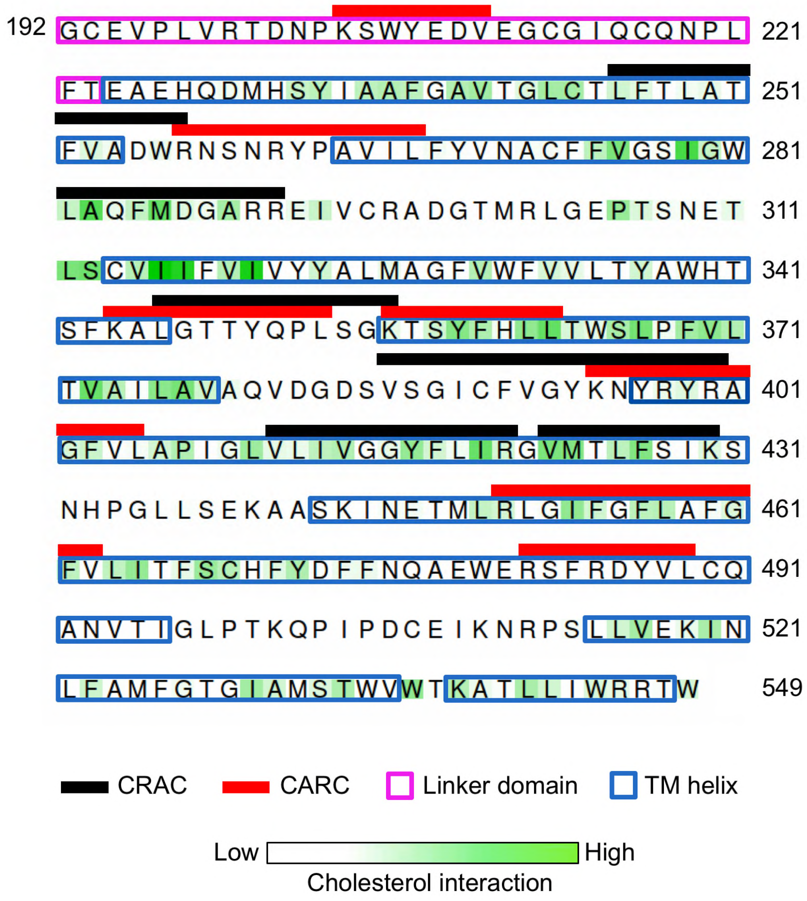

Figure S7. Co-localization of cholesterol contacts with putative cholesterol interaction motifs. Related to Figure 3.

The primary sequence of the simulated construct is shown, colored according to cholesterol interaction, from white (no contact) to green (high contact) using ALINE (Bond and Schuttelkopf, 2009). Contact data was extracted from a 100 µs CGMD simulation. The positions of the linker domain (magenta boxes) and transmembrane helices (blue boxes) are shown according to the DSSP predictions used to assign secondary structure in the simulations. The position of CRAC and CARC motifs are denoted by black and red bars, respectively.
