# Supplementary material for: Cholesterol Interaction Sites on the Transmembrane Domain of the Hedgehog Signal Transducer and Class F G Protein-Coupled Receptor Smoothened
Source: Structure. 2019 Mar 5;27(3):549–559.e2. doi: 10.1016/j.str.2018.11.003 (PMC6408332; doi:10.1016/j.str.2018.11.003)
Supplement: Document S2. Article plus Supplemental Information [file mmc4.pdf]

Structure

Theory

Cholesterol Interaction Sites on the Transmembrane Domain of the Hedgehog Signal Transducer and Class F G Protein-Coupled Receptor Smoothened

Graphical Abstract

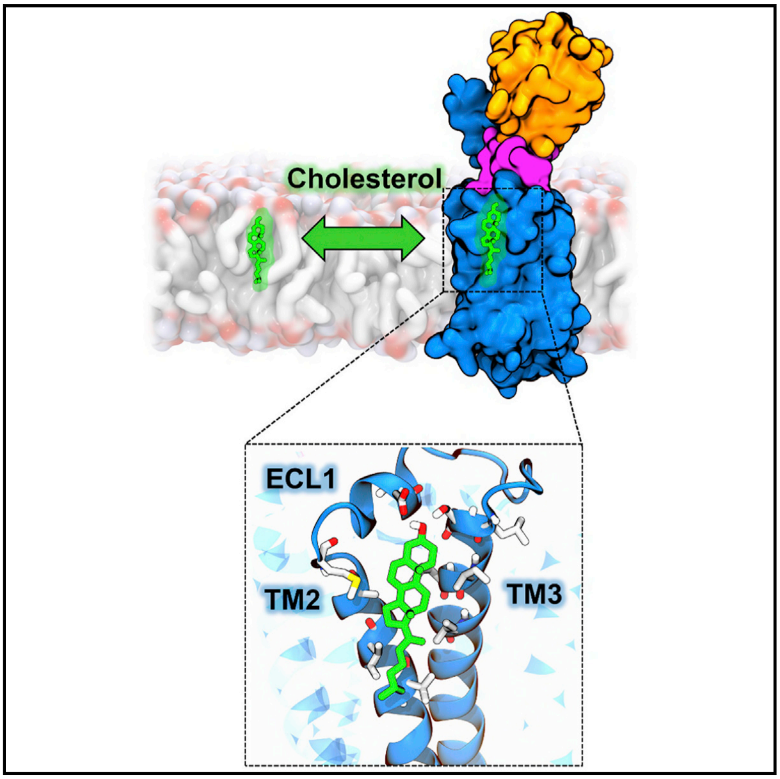

Highlights

- MD simulations predict a cholesterol binding site on the SMO transmembrane domain
- This site is distinct from those observed for class A GPCRs
- Potential of mean force calculations reveal the selectivity of sterol binding
- SMO forms strong encounter complexes with PIP_2 lipids

Authors

George Hedger, Heidi Koldsø, Matthieu Chavent, Christian Siebold, Rajat Rohatgi, Mark S.P. Sansom

Correspondence

mark.sansom@bioch.ox.ac.uk

In Brief

Hedger et al. use molecular simulations in model membranes to show interactions of the Smoothened GPCR with lipids. A cholesterol binding site is observed. Smoothened also forms strong encounter complexes with PIP_2 lipids. Together these results reveal possible mechanisms of lipid modulation of the function of Smoothened.

Hedger et al., 2019, Structure *27*, 549–559
March 5, 2019 © 2018 The Authors. Published by Elsevier Ltd.
https://doi.org/10.1016/j.str.2018.11.003

CellPress

CellPress

Cholesterol Interaction Sites on the Transmembrane Domain of the Hedgehog Signal Transducer and Class F G Protein-Coupled Receptor Smoothened

George Hedger,[1,5] Heidi Koldsø,[1,6] Matthieu Chavent,[2] Christian Siebold,[3] Rajat Rohatgi,[4] and Mark S.P. Sansom[1,7,*]
[1]Department of Biochemistry, University of Oxford, South Parks Road, Oxford OX1 3QU, UK
[2]Institut de Pharmacologie et de Biologie Structurale IPBS, Université de Toulouse, CNRS, UPS, Toulouse, France
[3]Division of Structural Biology, Wellcome Centre for Human Genetics, University of Oxford, Oxford OX3 7BN, UK
[4]Departments of Biochemistry and Medicine, Stanford University School of Medicine, Stanford, CA 94305, USA
[5]Present address: D. E. Shaw Research, 120 W. 45th St., 39th Fl., New York, NY 10036, USA
[6]Present address: Schrödinger, 120 W. 45th St., 17th Fl., New York, NY 10036, USA
[7]Lead Contact
*Correspondence: mark.sansom@bioch.ox.ac.uk
https://doi.org/10.1016/j.str.2018.11.003

SUMMARY

Transduction of Hedgehog signals across the plasma membrane is facilitated by the class F G-protein-coupled-receptor (GPCR) Smoothened (SMO). Recent studies suggest that SMO is modulated via interactions of its transmembrane (TM) domain with cholesterol. We apply molecular dynamics simulations of SMO embedded in cholesterol containing lipid bilayers, revealing a direct interaction of cholesterol with the TM domain at regions distinct from those observed in class A GPCRs. In particular the extracellular tips of helices TM2 and TM3 form a well-defined cholesterol interaction site. Potential of mean force calculations yield a free energy landscape for cholesterol binding. Alongside analysis of equilibrium cholesterol occupancy, this reveals the existence of a dynamic "greasy patch" interaction with the TM domain of SMO, which may be compared with previously identified lipid interaction sites on other membrane proteins. These predictions provide molecular-level insights into cholesterol interactions with a class F GPCR, suggesting potential druggable sites.

INTRODUCTION

The Smoothened (SMO) receptor is a critical component of the (Hh) signaling cascade, which controls a variety of key developmental processes, including human embryonic tissue patterning and regulation of adult stem cells (Briscoe and Thérond, 2013). Aberrant activation of SMO causes uncontrolled Hh signaling and a variety of cancers (Wu et al., 2017). As such SMO is of major academic and pharmaceutical interest, and is the target of two FDA-approved drugs for treating basal cell carcinomas, vismodegib (Dlugosz et al., 2012) and sonidegib (Burness, 2015).

SMO is a member of the Frizzled class of G-protein-coupled receptors (GPCRs) and is found at the plasma membrane. Its structural architecture consists of an extracellular cysteine-rich domain (CRD), stacked on top of a short linker domain (LD), and a hepta-helical transmembrane domain (7TMD) (Byrne et al., 2016; Zhang et al., 2017) (Figure 1). To date, 11 crystal structures containing the SMO 7TMD have been solved (Byrne et al., 2018), revealing structural similarity to the presumed inactive state of class A GPCRs (Wang et al., 2013). However, SMO exhibits <10% sequence identity to class A GPCRs, and is missing the canonical D[E]RY and NPxxY motifs implicated in the signaling mechanisms of class A receptors (Wang et al., 2013).

While the mechanism of SMO activation in the course of physiological Hh signaling has been a long-standing mystery, a number of recent studies have suggested that cellular cholesterol plays a central role in SMO activation and transduction of the Hh signal across the membrane (Blassberg et al., 2016; Byrne et al., 2018; Cooper et al., 2003; Huang et al., 2016; Luchetti et al., 2016; Myers et al., 2013). Reduced cellular cholesterol levels, as seen in Smith-Lemli-Opitz syndrome, or as produced when cells are treated with methyl-β-cyclodextrin, lead to decreased SMO activity and blunted Hh responses in target cells (Blassberg et al., 2016; Cooper et al., 2003). In addition, interesting synergistic effects on SMO function have been reported (Gordon et al., 2018) between statins, used to suppress cholesterol biosynthesis, and the SMO antagonist vismodegib, in the treatment of medulloblastoma. Further, oxysterols, hydroxylated metabolites of cholesterol, were shown to function as direct SMO agonists, suggesting that SMO could function as a sterol receptor (Nachtergaele et al., 2012).

The near full-length crystal structure of the protein revealed an extracellular cholesterol binding site located within the CRD (Byrne et al., 2016). Binding of cholesterol at this site was subsequently shown to be both necessary and sufficient for activation of SMO and Hh signaling (Huang et al., 2016; Luchetti et al., 2016). However, constitutively active truncations of SMO entirely lacking the CRD and SMO mutants that cannot bind sterols through the CRD (Blassberg et al., 2016; Briscoe and Thérond, 2013) still depend on the presence of membrane cholesterol

This is an open access article under the CC BY license (http://creativecommons.org/licenses/by/4.0/).

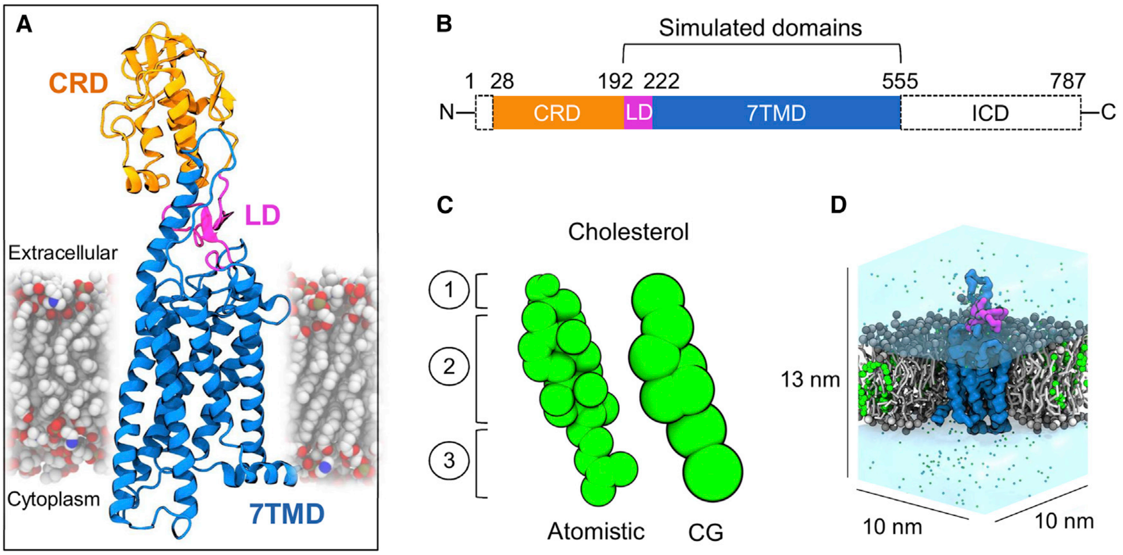

Figure 1. System Overview

(A and B) Molecular architecture (PDB: 5L7D) of the multi-domain human SMO protein, consisting of an extracellular CRD (orange), Linker domain (magenta), 7TM domain (blue), and structurally unresolved intracellular domain (ICD).

(C) Sphere representation of atomistic and CG Martini cholesterol, depicting the polar hydroxyl head group (1), hydrophobic cyclic ring system (2), and iso-octanyl tail (3).

(D) Cross-section through a CG system showing the simulated SMO construct (linker domain + 7TMD) embedded in a cholesterol (green) containing phosphatidylcholine (PC) lipid bilayer. Water is depicted as a transparent surface, and ions shown as sphere representations.

for their activity (Myers et al., 2017), suggesting that cholesterol may also regulate SMO activity through a second site within the TM. However at present no molecular level detail exists on the possible location of such an interaction.

Coarse-grained (CG) (Marrink et al., 2007) molecular dynamics simulations (Figure 1) enable the study of lipid interactions with integral membrane proteins (Hedger and Sansom, 2016). This approach provides access to the long simulation timescales and large system sizes required for thorough sampling of lipid-protein interaction space (Corradi et al., 2018). For example, this approach has previously been applied to predict the location of PIP_2 binding sites on Kir channels (Stansfeld et al., 2009), supported by a subsequently determined PIP_2-bound crystal structure (Hansen et al., 2011), and to characterize the structural, dynamic, and energetic aspects of cardiolipin interactions with the ADP/ATP translocase (Duncan et al., 2018; Hedger et al., 2016b), in agreement with available NMR and crystallographic data. More recently, the predicted locations of PIP_2 binding sites on class A GPCRs have been shown to agree with the results of site-directed mutagenesis and native mass spectrometry experiments (Yen et al., 2018). A number of other studies have compared the accuracy of the CG simulation approach to available experimental data on lipid binding for a variety of membrane proteins (Arnarez et al., 2013a, 2013b). In the case of cholesterol, the model recently identified both novel and established cholesterol binding sites on the adenosine A2A receptor (Rouviere et al., 2017), as well on as the dopamine transporter (Zeppelin et al., 2018), and Kir2.2 (Barbera et al., 2018). For a comprehensive review of cholesterol interactions studied by molecular dynamics simulations see (Grouleff et al., 2015), and for a comparative analysis of cholesterol interactions with a range of membrane proteins see (Lee, 2018).

Here we present multiscale simulations of human SMO embedded in a cholesterol-containing membrane environment (Figure 1D & Table 1). Cholesterol interactions are addressed via equilibrium CG simulations in both a simple two-component bilayer, and in a more complex *in vivo* mimetic membrane. We also calculate potentials of mean force to estimate the free energy of the SMO-cholesterol interaction in a bilayer environment. Our data predict a direct interaction of cholesterol with the TM of SMO, at defined locations.

RESULTS

All simulations were conducted using the GROMACS 4.6.5 (www.gromacs.org) simulation package (Hess et al., 2008). An overview of simulations performed is detailed in Table 1.

Cholesterol Interacts Directly with the SMO TM Domain at Defined Regions

The CG model of the 7TMD + LD of SMO were initially embedded in a PC:Chol (80%/20%) membrane (Table 1), with the cholesterol content chosen based on mammalian lipidomics estimates (Ingolfsson et al., 2014; van Meer et al., 2008). Eight separate systems with different initial lipid configurations were constructed, and used to initiate 8 × 10 μs of coarse-grained molecular dynamics (CGMD) simulations.

In all cases cholesterol approached the protein on a sub-microsecond timescale, forming direct interactions with the TM. The average molar composition of the first lipid interaction shell was 7.8 cholesterol and 26.7 PC molecules (22%

Table 1. Overview of the Simulations Performed

Description	Membrane Composition	Duration (μs)
7TMD and linker domain, CG	PC (80%) + Chol (20%)	8 × 10
7TMD and linker domain, CG	outer leaflet: PC (60%) + PE (15%) + Chol (25%) inner leaflet: PC (10%) + PE (40%) + PS (15%) + PIP_2 (10%) + Chol (25%)	8 × 10
7TMD and linker domain, CG, virtual site cholesterol (Melo et al., 2015)	PC (80%) + Chol (20%)	3 × 10
7TMD and linker domain, CG	PC (80%) + Chol (20%)	1 × 100
SMO 7TM and linker domain, AT	PC (80%) + Chol (20%)	3 × 0.2
7TMD PMF calculations, CG	PC (100%)	32 × 1 per PMF
7TMD and linker domain, CG	PC (100%)	3 × 10
Alternative conformation (Huang et al., 2018) of the 7TMD and linker domain, CG	PC (80%) + Chol (20%)	8 × 10

The box size for equilibrium simulations was 10 × 10 × 13 nm^3. The tail saturation pattern for phospholipids was 1-palmitoyl-2-oleoyl.

cholesterol content) over the simulated time course (Figure S1A). This indicates no significant annular enrichment in cholesterol compared with the bulk membrane (20% cholesterol content). Rather than form uniform interactions across the membrane-exposed surface in a non-specific fashion, cholesterol molecules were seen to localize at defined regions around the protein. Calculation of the time-averaged density for cholesterol around the protein revealed a particularly intriguing propensity for interaction with TM2/3 (Figure 2A). The extracellular portions of these helices (TM2/3e), together with ECL1, form a groove-like architecture on the protein surface into which a single cholesterol molecule slots (Figure 2B). Consistently high density at this site was observed across all eight simulation sites, independent of the starting configurations (Figure S1B). This contrasts with the more diffuse densities observed at other regions.

In order to assess the robustness of our observations of SMO-cholesterol interaction patterns in a simple two-component bilayer to changes in lipid composition, we repeated the initial 8 × 10 μs set of simulations in a five-component lipid bilayer containing PC, PE, PS, PIP_2, and cholesterol. These lipids, their distribution between leaflets, and their relative percentages were chosen to mimic a simplified *in vivo* plasma membrane composition (Table 1). No significant differences in cholesterol interactions were seen compared with the initial two-component (PC + cholesterol) membranes (Figure 2). This demonstrates that our simulation procedure reproducibly identifies a pattern of cholesterol interactions with SMO, especially the TM2/3e site, in two independent extensive ensembles of simulations with different lipid bilayer compositions.

To assess the molecular interactions giving rise to this density, the number of contacts formed between cholesterol and each residue of the protein were calculated over the simulated time course. Mapping these contacts onto the protein structure revealed cholesterol interaction hotspots on the membrane-exposed surface (Figures 3 and S2A). Within the TM2/3e site, the highest degree of cholesterol contact was formed with V276, I279, A283, M286, L312, S313, I316, I317, and I320 (Figure 3B). The majority of these contacts occurred with the hydrophobic moieties of cholesterol, while a degree of interaction was also seen for the head group hydroxyl with S313. The triad of isoleucine residues formed particularly high levels of interaction, a trend which has been observed for cholesterol binding sites across multiple GPCRs (Gimpl, 2016). Interestingly, although well-defined density was absent around TM4, the contact analysis coupled with visual inspection of the trajectories showed a moderate level of more dynamic interaction within this region. We explored the robustness of these predicted contacts by also using an alternative set of CG cholesterol parameters employing virtual sites (Melo et al., 2015), performing three independent replicates each of 10 μs duration. The same residue-by-residue cholesterol interaction pattern was observed compared with the standard parameter set (de Jong et al., 2013; Marrink et al., 2008) (Figure S2B).

Recently, two additional structures of *Xenopus laevis* SMO emerged, bound to cholesterol and to cyclopamine at the CRD (Huang et al., 2018). Both structures exhibited an alternative 7TMD conformation in which the intracellular portion of TM6 adopts an arrangement in which the intracellular tip moves outward by several Ångstroms compared with previous SMO structures (Byrne et al., 2016; Wang et al., 2013, 2014; Weierstall et al., 2014; Zhang et al., 2017). We subjected the 7TMD of the cyclopamine bound structure (PDB: 6D32) to 8 × 10 μs of CGMD, performed in the same manner as already described. The same TM2/3e binding site as seen in the human SMO structure was also observed in the alternative *X. laevis* structure (Figure S3B). Interactions at TM5 and TM6 were similar in both sets of simulations, exhibiting some degree of more transient interaction, but no stable localization as evidenced by the lack of well-defined cholesterol density in this region in both sets of simulation (Figure S6B). The rather more diffuse and weaker patterns of cholesterol density around TM5/6, compared with TM2/3e, were in both sets of simulations located at the intracellular end of TM5 and TM6, and at the extracellular end of TM6. Interestingly in the case of the *X. laevis* simulations the balance of contacts between TM5/6 on the intracellular side skewed more toward TM6 rather than TM5; however, this had little impact on the propensity of cholesterol to localize at this region (Figure S6B). Notably, we did not observe spontaneous entry of cholesterol into the core of the protein between TM5/6 as has been proposed (Huang et al., 2018).

Long Timescale Simulation of Cholesterol Dynamics

To enable assessment of the dynamics of the interaction and better test system convergence, we extended one CG simulation

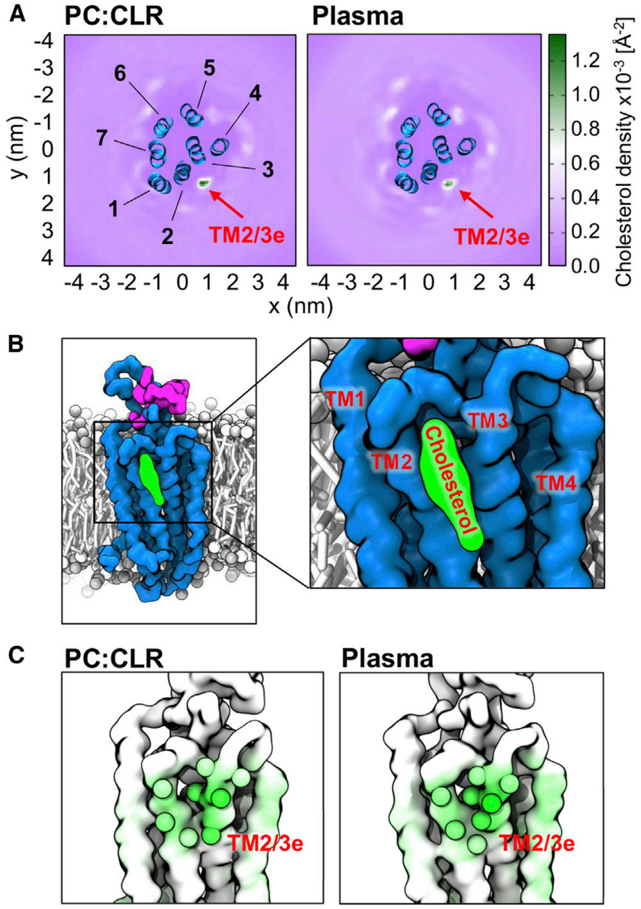

Figure 2. Cholesterol Forms Direct Interactions with SMO and Localizes to Defined Regions

(A) 2D time-averaged density projections for membrane cholesterol around SMO in a simple two-component membrane composition (left) and a plasma membrane mimetic (right).

(B) Final snapshot (t = 10 μs) from a simulation showing cholesterol bound at the TM2/3e site.

(C) View onto the TM2/3e site with simulation snapshots of the protein colored from white (no contact) to green (high contact) according to the degree of interaction with cholesterol. Data for both membrane compositions were averaged over eight independent CGMD simulations, each of 10 μs duration, initiated from different random lipid configurations. See Table 1 for further details of the membrane compositions employed.

from 10 to 100 μs. No significant evolution in cholesterol interaction patterns were observed on this long timescale compared with the ensemble of shorter simulations (Figure S3A), again indicating convergence of the system properties under consideration.

Calculation of the time-dependent occupancy of the TM2/3e site revealed that the site remained occupied by a cholesterol molecule for close to the entire duration of the 100-μs simulation (Figure 4A). This high level of occupancy could result either from extended binding of a single cholesterol molecule, or from rapid exchange events between multiple different molecules. We assessed this question by decomposing the occupancy data to form an interaction matrix for individual cholesterol molecules, showing the occupancy between each individual cholesterol index in the simulation and the TM2/3e site (Figure 4B). The dynamic nature of the interaction is apparent, with exchange between different cholesterol molecules at the TM2/3e site on a sub-microsecond timescale (Figure 4C) (Video S1), leading to exhaustive sampling by all 54 molecules over the course of the 0.1-ms simulation. Binding events ranged from transient interactions in the order of tens of nanoseconds, through to extended interactions of 1 μs or more. Visual inspection of the longest binding event (7 μs duration) revealed that, even within extended interaction events, the bound cholesterol molecule was dynamically localized and frequently rotated around its long axis, alternately exposing its rough β face to the membrane and binding site. This observation of a dynamic interaction with a "greasy patch," as opposed to "rigid binding," concurs with the findings of Lyman and colleagues for the adenosine A2A receptor (Rouviere et al., 2017), and a range of other cholesterol binding membrane proteins (Grouleff et al., 2015), and may in turn correlate with the absence of well-defined cholesterol density at this region in available crystal structures of SMO (Byrne et al., 2016; Wang et al., 2013).

All-Atom Simulations Also Demonstrate a Direct Cholesterol Interaction and Reveal Additional Molecular Details

To better investigate the atomic level details of the interaction, three snapshots from the CG simulations with cholesterol bound at TM2/3e were converted to atomic resolution. Each simulation was equilibrated for 10 ns with position restraints on the protein, before being run for 200 ns of unbiased molecular dynamics. The protein remained stable with Cα root-mean-square deviation (RMSD) of the TM helices plateauing at ~ 0.2 nm for all three repeats (Figure S4). The predominant structural fluctuations were seen in disordered loop regions, and particularly the long ECL3 connecting TM6 and TM7. These observations agree well with our previous atomistic simulations of the full-length SMO construct (Byrne et al., 2016). In all three simulations the cholesterol molecule at site TM2/3e remained bound, undergoing frequent rotation about its long axis, alternately exposing the methyl groups of its rough β face to the membrane and binding site, as observed in the CG simulations (Figure 5A) (Video S2). In two cases the cholesterol molecule underwent partial dissociation (Figure 5B, red arrows) before re-binding to adopt its previous orientation, emphasizing the dynamic nature of the interaction. Interestingly, E292, not identified in the CG simulations, was also seen to form interactions with the hydroxyl group of the bound cholesterol, and the adjacent S313 (Figure 5C). This additional observation underscores the value in adopting a serial multiscale approach (Stansfeld and Sansom, 2011) in the investigation of lipid-protein interactions.

Potential of Mean Force Calculations for Lateral Cholesterol Interaction

To assess the strength and selectivity of cholesterol interaction at TM2/3e we calculated the potential of mean force (PMF) for the lateral interaction of membrane cholesterol with the site. The PMF describes the change in free energy between two species along a particular reaction coordinate, and is derived from the probability distribution along this coordinate (Roux, 1995). A steered MD simulation was performed in which a force was

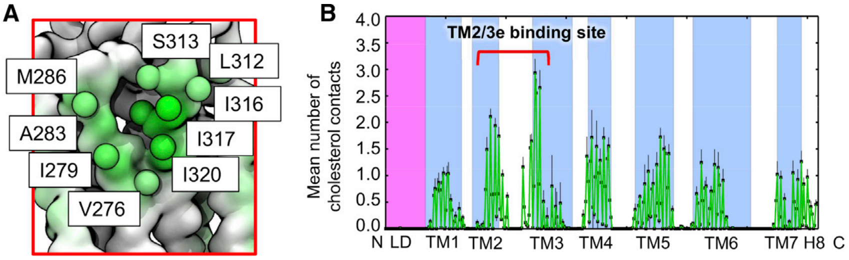

Figure 3. Per residue Time-Averaged Cholesterol Contacts with SMO

(A) Zoom-in on the putative TM2/3e cholesterol binding pocket with binding site residues labeled and depicted as spheres, colored from white (no contact) to green (high contact) according to the mean number of contacts formed with cholesterol.

(B) Global view of the mean number of cholesterol contacts for each residue of the protein, with the standard deviation (n = 8) denoted by black error bars. The linker domain and transmembrane helices are delineated by magenta and blue boxes. Contacts were calculated using a 6 Å distance cutoff to define ''contact'', based on the radial distribution function for CG Martini lipid-protein interactions.

applied to pull the bound cholesterol molecule away from its binding site into the bulk membrane. This generated a lateral 1D reaction coordinate (r) perpendicular to the protein surface, ranging from the bound to unbound state. Umbrella sampling was then applied to calculate the free energy profile along this coordinate, with the reaction coordinate r defined as the distance between the center-of-mass of the TM2/3e binding site and the cholesterol molecule.

The profile uncovered a maximal well depth of ca. -10 kJ/mol at TM2/3e, for both the standard (Marrink et al., 2008) and virtual site (Melo et al., 2015) cholesterol parameters (Figure 6). In contrast, repeating the calculation for a separate site on the intracellular portion of the protein (the cholesterol consensus motif or CCM, which has been suggested to bind cholesterol in class A GPCRs [Hanson et al., 2008]) which is not predicted to bind cholesterol from our equilibrium simulations, yielded a well depth <2.5 kJ/mol ($\sim$RT), indicating no significant interaction at this site (Figure S5A). Repeating the same calculation at TM2/3e for PC, PE, and PS lipids, which comprise a significant portion of plasma membrane lipids (van Meer et al., 2008), likewise showed no significant interaction, demonstrating the selectivity of the site for cholesterol (Figure 6). These observations are consistent with the results of equilibrium CGMD simulations performed in PC-only membranes, which, in the absence of cholesterol, exhibit no specific binding of phospholipids to this site (Figure S6A).

SMO Binds PIP_2 Lipids

A number of studies (e.g., Dawaliby et al., 2016) have suggested that phospholipids may allosterically regulate GPCRs. We therefore simulated SMO in a five-component lipid mixture mimicking an *in vivo* plasma membrane in composition and distribution (see previous sections for analysis of cholesterol interactions in this environment). These simulations revealed a high degree of interaction with negatively charged PIP_2 lipids (Figure 7). This is of particular interest, as PIP_2 has recently been shown to act as a positive allosteric modulator of class A GPCRs, forming ''encounter complexes'' enhancing G protein coupling by simultaneously contacting both structures as a ''bridge'' or ''molecular glue'' (Yen et al., 2018). The interaction of PIP_2 with SMO occurred at defined regions on the intracellular portion of the protein, with multi-valent interactions predominately mediated via binding of the tri-phosphorylated headgroups of PIP_2 to clusters of basic protein side chains (R257, K344, K356, K539, R546, and R547). The acyl tails formed comparatively few interactions with SMO. This is in keeping with observations from a range of other studies of specific anionic lipid-protein interactions (e.g., Barbera et al., 2018; Duncan et al., 2018; Ruprecht et al., 2014). This observation is likely to prove particularly intriguing should the binding of SMO to intracellular partners, such as G proteins, be structurally rationalized in the future. Furthermore, in an *in vivo* context, we note that SMO is enriched near the base of primary cilia, a zone which contains high levels of PIP_2. Ciliary phosphoinositides have been shown to regulate Hh signaling. Mutations in a 5-position phosphatase (Inpp5e) lead to alterations in the distribution of ciliary PIP_2 and cause Joubert's syndrome, a human ciliopathy characterized by impaired Hh signaling and human birth defects (Bielas et al., 2009; Chávez et al., 2015; Garcia-Gonzalo et al., 2015; Nakatsu, 2015). The simulation-based observation of direct PIP_2 binding to defined regions of SMO is therefore of especial interest.

DISCUSSION

We observe direct interactions of cholesterol with the 7TMD of human SMO using molecular dynamics simulations at a range of resolutions, timescales, parameter sets, and membrane compositions. This is particularly intriguing given the recent discovery of a functional dependence of the truncated 7TMD of SMO on membrane cholesterol (Myers et al., 2017), as well as longstanding pathophysiological observations made in human developmental disorders (Blassberg et al., 2016; Cooper et al., 2003), and the emerging suggestion that statins may synergize with the effects of vismodegib on SMO activity (Gordon et al., 2018). The identification of a well-defined cholesterol binding site located at TM2/3e is especially interesting. This cholesterol interaction is within the extracellular leaflet of the membrane. Recent structures of Patched1 (Ptch1) (Gong et al., 2018; Qi et al., 2018a, 2018b; Zhang et al., 2018) identify a potential interaction site between Ptch1 and cholesterol in a region that is also located in the extracellular leaflet. While the mechanism of Ptch1 modulation of SMO remains uncertain, this suggests the view that Ptch1 may regulate SMO via extracellular leaflet cholesterol (Liu et al., 2017; Sommer and Lemmon, 2018).

The cholesterol binding site at TM2/3e is distinct from the more transient interactions with the rest of the protein surface, as measured by density projections, contact analysis, analysis of occupancy dynamics, as well as free energy calculations. This site was consistently observed across multiple timescales, parameter sets, membrane compositions, and resolutions. In all cases cholesterol adopted a dynamic binding mode within this site, both undergoing frequent rapid exchange with bulk

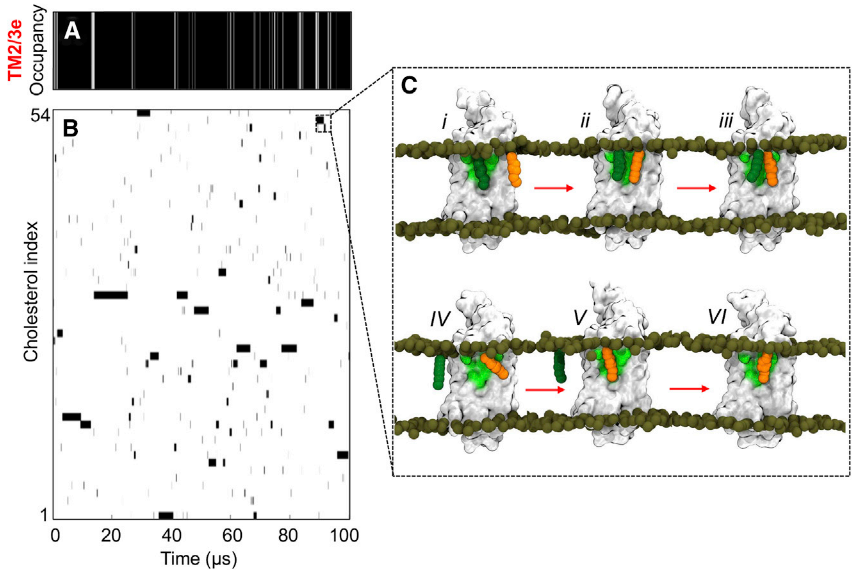

Figure 4. Dynamics of Cholesterol Interaction within Long-Timescale MD

(A) Occupancy of the TM2/3e site over time, with: black, occupied, and white, unoccupied.

(B) Interaction matrix showing the occupancy data for each individual cholesterol molecule with TM2/3e. This data shows that the TM2/3e site is occupied for almost the entire duration of the 100 μs simulation, and that this is due to multiple rapid exchanges between different cholesterol molecules rather than a single long-timescale binding of one cholesterol.

(C) A series of snapshots are shown (right) depicting an exchange event between two cholesterol molecules (orange, dark green). Occupancy data were calculated as previously described by us (Chavent et al., 2018) using a distance cutoff of 8 Å between the centre-of-mass of the TM2/3e site and the centre-of-mass of each cholesterol molecule, in keeping with (Arnarez et al., 2013b; Hedger et al., 2016a).

membrane cholesterol, and undergoing rotation about its long axis while bound. This is consistent with the results of PMF calculations at this site, which yielded a well depth of −10 kJ/mol. These observations point to a greasy patch model for cholesterol binding to SMO, as observed for the adenosine A_{2A} receptor (A2aR) (Rouviere et al., 2017) and the dopamine transporter (Zeppelin et al., 2018).

In this context, it is useful to compare our estimates of the strength of SMO/cholesterol interactions with those obtained from other simulation studies of GPCRs, while bearing in mind the different methodologies and bilayer compositions employed in the various studies. Thus, from analysis of extended (50 μs) equilibrium CG simulations of the β_2AR and A2aR in a cholesterol-containing membrane, Genheden et al. (2017) estimated free energies of interaction of the order of −10 to −15 kJ/mol. From equilibrium (2 × 0.8 μs) atomistic MD simulations of the A2aR, Lee and Lyman (2012) estimated free energies of – 3 to −5 kJ/mol. A comparable analysis of our equilibrium CG simulations yielded a free energy of interaction of −6 kJ/mol. Given the detailed differences between the various studies, a conservative interpretation would be that cholesterol interactions with GPCRs are of the order of −5 to −10 kJ/mol, in contrast with estimated free energies of −10 to −40 kJ/mol for the interactions of a range of different membrane proteins with anionic phospholipids calculated by the same approach (see [Arnarez et al., 2016; Arnarez et al., 2013b; Domański et al., 2017; Domicevica et al., 2018; Gu et al., 2017; Hedger et al., 2016a; Hedger et al., 2016b] and Table S1).

A number of cholesterol interaction sites have been determined for class A GPCRs (Sengupta and Chattopadhyay, 2015), the most well established of which is perhaps the CCM suggested for the β_2AR (Hanson et al., 2008). Our observations point to distinct cholesterol interaction patterns on SMO. This is perhaps not surprising. Although SMO, a class F GPCR, bears a high degree of structural similarity to its class A counterparts, it is distant in sequence with a sequence identity <10% (Wang et al., 2013). Additionally, an emerging pattern from the analysis of class A GPCR cholesterol interactions is that these are often receptor specific, with limited sequence conservation (Gimpl, 2016). We did not observe any significant cholesterol interaction at the region of SMO corresponding to the CCM of the β_2AR. This is perhaps unsurprising as the equivalent involved residues in SMO are differently distributed in space compared with those suggested for β_2AR (Hanson et al., 2008), resulting in an arrangement for which it is difficult to explain how cholesterol could simultaneously contact the key residues of the motif. Intriguingly the TM2/3e site contained a triad of isoleucine residues, which formed the highest levels of cholesterol contact among all other SMO residues. This shows a striking correlation with earlier findings (Gimpl, 2016), in which isoleucine residues were shown to be heavily enriched in the cholesterol binding sites of GPCRs determined by X-ray crystallography.

Regarding cholesterol interaction sites more broadly, CRAC and CARC motifs have been suggested in some cases to form regions more conducive toward cholesterol binding (Epand, 2006; Fantini and Barrantes, 2013; Li and Papadopoulos, 1998). The general nature of the definition of these motifs results in their presence in most membrane proteins. Indeed, the simulated SMO construct contains a total of 13 of these motifs. However, most of these can be discounted as candidates simply because they are not membrane exposed, or their geometry is otherwise such that it is difficult to rationalize how cholesterol would bind. Comparison of the location of these motifs with the degree of per residue cholesterol contacts extracted from the CGMD simulations (Figure S7), revealed a degree of co-localization with the intracellular ends of TM4 and TM5. These regions exhibit lower levels of cholesterol occupancy and do not yield well-defined cholesterol density compared with TM2/3e. Nonetheless direct interaction is observed and the co-localization with the CRAC/CARC motifs is interesting.

Regarding lipid interactions in more complex multi-component membranes, the reproducibility of cholesterol interaction patterns in a plasma membrane mimetic is especially encouraging. This reproducibility suggests, in this case, the absence of competition effects with other major plasma membrane lipids, as also supported by the PMF calculations. The absence of such

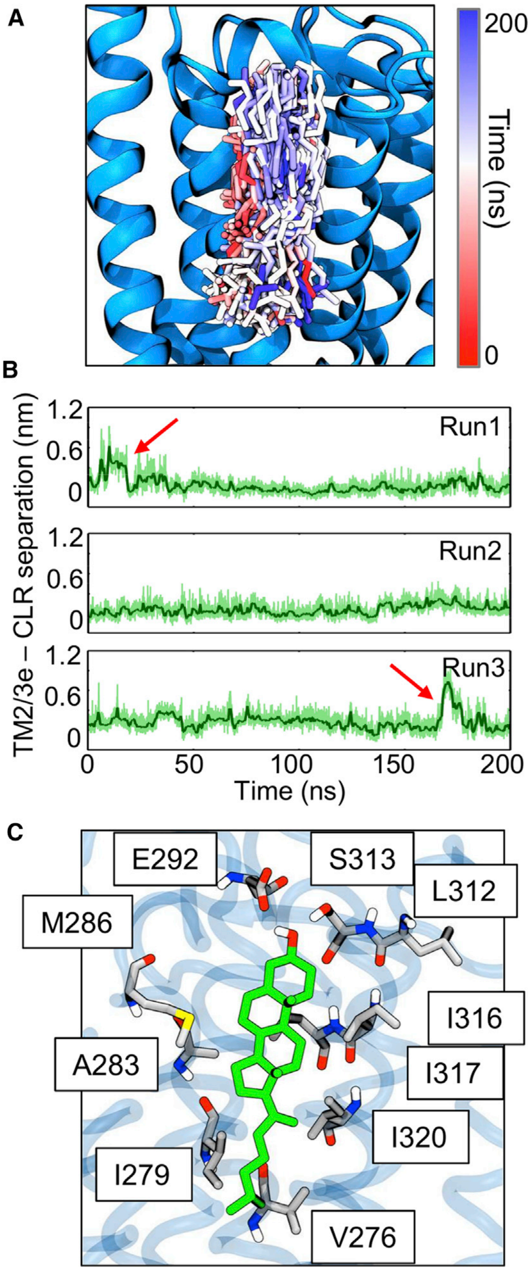

Figure 5. Cholesterol Interactions within All-Atom Simulations

(A) Snapshot of SMO embedded in a lipid bilayer. A total of 600 simulation snapshot structures of cholesterol (stick representation) at the TM2/3e binding pocket are depicted. Snapshots of the cholesterol structure were taken every 1 ns over the course of each 200 ns simulation, across all three repeats. Each of these structures is colored according to the corresponding simulation time on the RWB colorscale shown, thus structures at the start of each simulation are colored red, and those at the end in blue.

(B) Distance between the centre-of-mass of cholesterol and the TM2/3e binding pocket.

(C) Final simulation snapshot (t = 200 ns) showing the arrangement of key binding site residues.

effects is perhaps intuitively more likely for cholesterol, which is disparate in structure, physiochemical properties, and membrane insertion depth compared with its phospholipid counterparts, and may thus be expected to interact with embedded proteins via rather different modes. The additional observation of the formation of a PIP_2-SMO encounter complex in these membranes, similar to simulation/native mass spectrometry-based observations for class A GPCRs (Yen et al., 2018), is also intriguing and raises the prospect of potential involvement of PIP_2 in modulation of SMO binding to putative intracellular interaction partners.

These results provide a testable hypothesis as to the manner of cholesterol interaction with SMO. We propose a number of routes for experimental testing of our observations. In the first instance, these predictions could be tested by site-directed mutagenesis coupled to subsequent functional assays. Such functional assays could either be conducted using signaling assays in cells (Luchetti et al., 2016), or indeed in minimal *in vitro* reconstituted nanodisc systems, where the activity of the truncated 7TMD has been shown to depend on cholesterol (Myers et al., 2017). Secondly, native mass spectrometry has shown recent tremendous utility in probing the specific binding of lipids to membrane proteins (Gupta et al., 2018; Laganowsky et al., 2014). Coupling this approach to a mutagenesis strategy could provide an exciting route to identify putative cholesterol interaction regions. A note of caution, however, that some uncertainty remains as to the ability of native mass spectrometry to identify weak binding lipid species, including cholesterol. Most cases to date have focused on rigid high-energy binding of species such as PIP (Laganowsky et al., 2014), PE (Patrick et al., 2018), and cardiolipin (Gupta et al., 2017), which may be expected to better survive detergent solubilization. This potential caveat is true also for crystallographic methods (Yeagle, 2014). In both of these cases it is worth highlighting also that mutagenesis of GPCRs to identify lipid binding is a non-trivial undertaking. One must be cognizant of the possible need for simultaneous mutation of clusters of binding site residues in order to evoke sufficient perturbation of the lipid binding site and preventing competition effects from neighboring residues (Yen et al., 2018), while at the same time remaining cognizant to the sensitivity of GPCR expression and folding to introduced perturbations. Careful choice of mutants and extensive controls are likely necessary. Thirdly, an exciting approach to identify cholesterol interaction sites is photo-sensitive chemical crosslinking, also referred to as "click" assays (Hulce et al., 2013), which utilize photo-reactive cholesterol analogs to trap the interaction before subsequent mass spectrometry analysis. It is important to consider that, while (1) tests whether the identified regions influence SMO signaling activity, (2) and (3) focus on testing simply whether cholesterol binds at particular sites, or not. It is possible of course that cholesterol interaction sites could have a range of biological functions besides influencing SMO signaling activity, including e.g., modulating lateral interactions with other biomolecules as has been seen for other lipid-protein interactions, and effects on stability. Both of which have been observed for other GPCRs (Prasanna et al., 2014; Zocher et al., 2012).

Limitations

Accurate interpretation of our predictions requires a discussion of the limitations of the approaches used and currently available experimental data on which to base our model. The Martini model involves an inherent simplification of chemical detail (Marrink

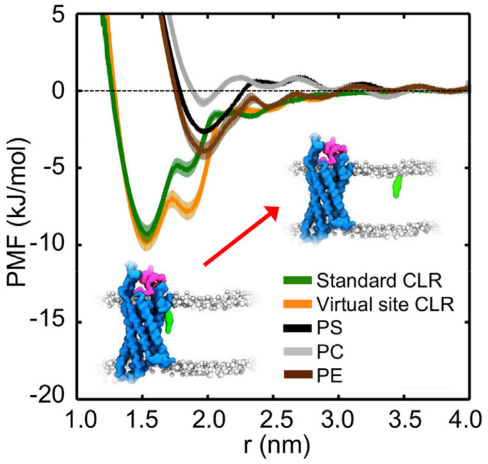

Figure 6. Potential of Mean Force Calculations for Lateral Lipid Interaction

PMF profiles for cholesterol interactions at the TM2/3e site. A profile obtained using the standard Martini cholesterol parameters (green) is compared with a profile obtained using the new virtual model (orange); and to the phospholipids PS (black), PC (gray), and PE (brown). Insets depict simulation snapshots of bound and unbound cholesterol. The shaded areas behind each curve indicates the standard deviation estimated from bootstrapping.

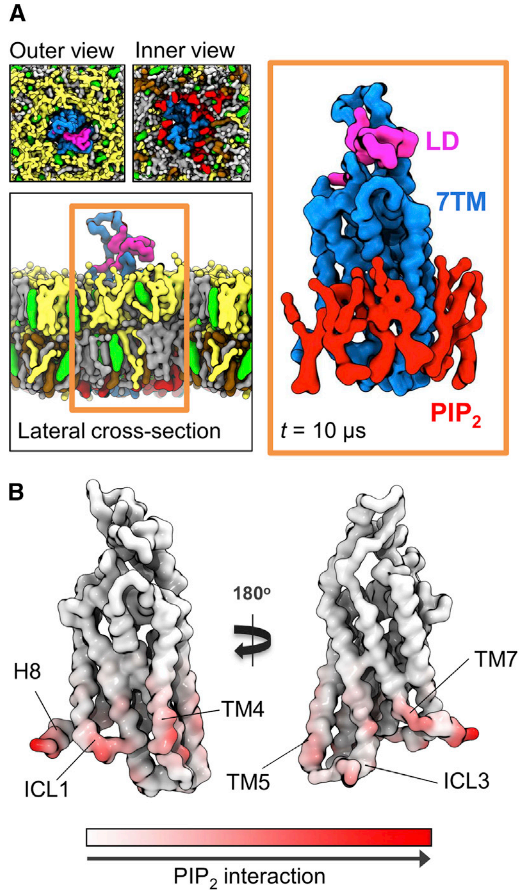

Figure 7. Plasma Membrane Mimetic Simulations

(A) Final snapshots of SMO embedded in a PC (yellow), PE (gray), PS (ochre), PIP_2 (red), and cholesterol (green) membrane. Protein and lipids are rendered as surfaces. The formation of a PIP_2-SMO complex (orange box) is apparent. Water and ions have been omitted for clarity. Other lipid species have been omitted from the right-hand image of the PIP_2 encounter complex.

(B) Mean number of PIP_2 contacts mapped onto structure, with each residue colored from white (no contact) to red (high contact). The predominant mode of PIP_2 contact with SMO can be seen to occur in the head group region, with the acyl tails forming comparatively less contact. Contacts were calculated over an ensemble of 8 × 10 μs of CGMD, using a 6 Å distance cutoff. See the STAR Methods for further details of contact analysis. Each simulation was initiated from a different random distribution of lipids around the protein.

et al., 2007). This is a tradeoff made to access the time and length scales required for sufficient sampling of lipid-protein configurational space. Obtaining converged calculations of this nature in all-atom detail remains challenging without the use of specialized bespoke supercomputing resources (Shaw et al., 2008), and alternative enhanced sampling approaches (Domański et al., 2018). Importantly, we consider only one conformational state of the protein, for which structures have been determined. How might the location of cholesterol interaction sites mechanistically affect function? This is a challenging question to address at this time. In class A GPCRs the major conformational transition between inactive and active occurs at the TM5, ICL3, TM6 interface (Dror et al., 2011). However significant conformational changes are possible at other regions such as ICL2 in the κ-opioid receptor (Che et al., 2018). The extensibility of these observations to class F GPCRs remains uncertain. Should alternative conformational states of SMO emerge either from further structures and/or long timescale all-atom MD, it would be extremely interesting to re-visit cholesterol interactions and assess any possible deviations in interaction pattern.

Regarding our failure to observe cholesterol entry into the core of SMO (Huang et al., 2018) in the *X. laevis* simulations, it is prudent to state that the necessary application of an ElNeDyn network (Periole et al., 2009) to SMO prohibits significant deviations in conformation, while preserving local dynamics. Thus we test whether cholesterol could enter from the membrane for this particular conformation state. It is possible, of course, that conformational changes not captured by our model could be required to enable entry. Atomistic simulations would be required to computationally assess such a process.

Despite these limitations, the approaches discussed have achieved significant success in identifying a range of lipid interaction sites on membrane proteins, controlled and tested against experimental data including mass spectrometry (Gupta et al., 2017; Liko et al., 2016; Yen et al., 2018), crystallographic (Arnarez et al., 2013a; Schmidt et al., 2013; Van Eerden et al., 2017; Zeppelin et al., 2018), NMR spectroscopy (Duncan et al., 2018; Hedger et al., 2016b), and mutational functional data (Hedger et al., 2015, 2016a; Stansfeld et al., 2009).

Conclusions

These data provide key molecular level detail on the location and modes of direct cholesterol interaction with the 7TM domain of SMO, a class F GPCR of significant pharmaceutical interest, with an emerging intricate functional relationship with cholesterol. Identification and molecular level characterization of these sites is a first step toward understanding the mechanistic implications, and possible routes to therapeutic intervention via the

design of small molecule mimetics, or the targeted control of cholesterol metabolism (Gordon et al., 2018).

STAR★METHODS

Detailed methods are provided in the online version of this paper and include the following:

- KEY RESOURCES TABLE
- CONTACT FOR REAGENT AND RESOURCE SHARING
- METHOD DETAILS
    - SMO Model Building
    - Coarse-Grained Simulations
    - Analysis
    - Contact Analysis
    - Density Analysis
    - Potential of Mean Force Calculations
    - Atomistic Simulations
- DATA AND SOFTWARE AVAILABILITY

SUPPLEMENTAL INFORMATION

Supplemental Information includes seven figures, one table, and two videos and can be found with this article online at https://doi.org/10.1016/j.str.2018.11.003.

ACKNOWLEDGMENTS

We thank S.L. Rouse (Imperial College London, UK), B. Schiøtt (Aarhus University, Denmark), J. Schnell (University of Oxford, UK), and members of the M.S.P.S. laboratory. G.H. acknowledges the Medical Research Council for PhD and postdoctoral funding. Research in the M.S.P.S. laboratory is supported by the Wellcome Trust, BBSRC, and EPSRC. This project made use of time on ARCHER granted via the UK High-End Computing Consortium for Biomolecular Simulation supported by EPSRC (grant no. EP/L000253/1). C.S. is funded by Cancer Research UK (C20724/A14414) and the European Research Council (647278). R.R. is supported by grants GM106078 and GM118082 from the National Institutes of Health.

AUTHOR CONTRIBUTIONS

G.H. set up, performed, and analyzed the simulations. G.H., C.S., R.R., and M.S.P.S. designed the research project. H.K. and M.C contributed analysis tools. G.H. and M.S.P.S. wrote the manuscript with contributions from all authors.

DECLARATION OF INTERESTS

The authors declare no competing interests.

Received: August 3, 2018
Revised: September 28, 2018
Accepted: November 2, 2018
Published: December 27, 2018

REFERENCES

Arnarez, C., Marrink, S.J., and Periole, X. (2013a). Identification of cardiolipin binding sites on cytochrome c oxidase at the entrance of proton channels. Sci. Rep. *3*, 1263.

Arnarez, C., Marrink, S.J., and Periole, X. (2016). Molecular mechanism of cardiolipin-mediated assembly of respiratory chain supercomplexes. Chem. Sci. *7*, 4435–4443.

Arnarez, C., Mazat, J.-P., Elezgaray, J., Marrink, S.-J., and Periole, X. (2013b). Evidence for cardiolipin binding sites on the membrane-exposed surface of the cytochrome bc1. J. Am. Chem. Soc. *135*, 3112–3120.

Barbera, N., Ayee, M.A.A., Akpa, B.S., and Levitan, I. (2018). Molecular dynamics simulations of Kir2.2 interactions with an ensemble of cholesterol molecules. Biophys. J. https://doi.org/10.1016/j.bpj.2018.07.041.

Berendsen, H.J.C., Postma, J.P.M., van Gunsteren, W.F., DiNola, A., and Haak, J.R. (1984). Molecular dynamics with coupling to an external bath. J. Chem. Phys. *81*, 3684–3690.

Bielas, S.L., Silhavy, J.L., Brancati, F., Kisseleva, M.V., Al-Gazali, L., Laszlo, S., Bayoumi, R.A., Zaki, M.S., Abdel-Aleem, A., Rosti, R.O., et al. (2009). Mutations in INPP5E, encoding inositol polyphosphate-5-phosphatase E, link phosphatidyl inositol signaling to the ciliopathies. Nat. Genet. *41*, 1032–U1108.

Blassberg, R., Macrae, J.I., Briscoe, J., and Jacob, J. (2016). Reduced cholesterol levels impair smoothened activation in Smith–Lemli–Opitz syndrome. Hum. Mol. Genet. *25*, 693–705.

Briscoe, J., and Thérond, P.P. (2013). The mechanisms of Hedgehog signalling and its roles in development and disease. Nat. Rev. Mol. Cell Biol. *14*, 416.

Burness, C.B. (2015). Sonidegib: first global approval. Drugs *75*, 1559–1566.

Bussi, G., Donadio, D., and Parrinello, M. (2007). Canonical sampling through velocity rescaling. J. Chem. Phys. *126*, 014101.

Byrne, E.F.X., Luchetti, G., Rohatgi, R., and Siebold, C. (2018). Multiple ligand binding sites regulate the Hedgehog signal transducer Smoothened in vertebrates. Curr. Opin. Cell Biol. *51*, 81–88.

Byrne, E.F.X., Sircar, R., Miller, P.S., Hedger, G., Luchetti, G., Nachtergaele, S., Tully, M.D., Mydock-McGrane, L., Covey, D.F., Rambo, R.P., et al. (2016). Structural basis of smoothened regulation by its extracellular domains. Nature *535*, 517–522.

Chavent, M., Karia, D., Kalli, A.C., Domański, J., Duncan, A.L., Hedger, G., Stansfeld, P.J., Seiradake, E., Jones, E.Y., and Sansom, M.S.P. (2018). Interactions of the EphA2 kinase domain with PIPs in membranes: implications for receptor function. Structure *26*, 1025–1034.e2.

Chávez, M., Ena, S., Van Sande, J., de Kerchove d'Exaerde, A., Schurmans, S., and Schiffmann, S.N. (2015). Modulation of ciliary phosphoinositide content regulates trafficking and sonic hedgehog signaling output. Dev. Cell *34*, 338–350.

Che, T., Majumdar, S., Zaidi, S.A., Ondachi, P., McCorvy, J.D., Wang, S., Mosier, P.D., Uprety, R., Vardy, E., Krumm, B.E., et al. (2018). Structure of the nanobody-stabilized active state of the kappa opioid receptor. Cell *172*, 55–67.e15.

Cooper, M.K., Wassif, C.A., Krakowiak, P.A., Taipale, J., Gong, R., Kelley, R.I., Porter, F.D., and Beachy, P.A. (2003). A defective response to Hedgehog signaling in disorders of cholesterol biosynthesis. Nat. Genet. *33*, 508.

Corradi, V., Mendez-Villuendas, E., Ingólfsson, H.I., Gu, R.-X., Siuda, I., Melo, M.N., Moussatova, A., DeGagné, L.J., Sejdiu, B.I., Singh, G., et al. (2018). Lipid–protein interactions are unique fingerprints for membrane proteins. ACS Cent. Sci. *4*, 709–717.

Dawaliby, R., Trubbia, C., Delporte, C., Masureel, M., Van Antwerpen, P., Kobilka, B.K., and Govaerts, C. (2016). Allosteric regulation of G protein-coupled receptor activity by phospholipids. Nat. Chem. Biol. *12*, 35–39.

de Jong, D.H., Singh, G., Bennett, W.F.D., Arnarez, C., Wassenaar, T.A., Schäfer, L.V., Periole, X., Tieleman, D.P., and Marrink, S.J. (2013). Improved parameters for the Martini coarse-grained protein force field. J. Chem. Theory Comput. *9*, 687–697.

Dlugosz, A., Agrawal, S., and Kirkpatrick, P. (2012). Vismodegib. Nat. Rev. Drug Discov. *11*, 437.

Domański, J., Hedger, G., Best, R., Stansfeld, P.J., and Sansom, M.S.P. (2017). Convergence and sampling in determining free energy landscapes for membrane protein association. J. Phys. Chem. B *121*, 3364–3375.

Domański, J., Sansom, M.S.P., Stansfeld, P.J., and Best, R.B. (2018). Balancing force field protein-lipid interactions to capture transmembrane helix-helix association. J. Chem. Theory Comput. *14*, 1706–1715.

Domicevica, L., Koldsø, H., and Biggin, P.C. (2018). Multiscale molecular dynamics simulations of lipid interactions with P-glycoprotein in a complex membrane. J. Mol. Graph. Model. *80*, 147–156.

Dror, R.O., Arlow, D.H., Maragakis, P., Mildorf, T.J., Pan, A.C., Xu, H., Borhani, D.W., and Shaw, D.E. (2011). Activation mechanism of the β_2-adrenergic receptor. Proc. Natl. Acad. Sci. U S A *108*, 18684–18689.

Duncan, A.L., Ruprecht, J.J., Kunji, E.R.S., and Robinson, A.J. (2018). Cardiolipin dynamics and binding to conserved residues in the mitochondrial ADP/ATP carrier. Biochim. Biophys. Acta *1860*, 1035–1045.

Epand, R.M. (2006). Cholesterol and the interaction of proteins with membrane domains. Prog. Lipid Res. *45*, 279–294.

Essmann, U., Perera, L., Berkowitz, M.L., Darden, T., Lee, H., and Pedersen, L.G. (1995). A smooth particle mesh Ewald method. J. Chem. Phys. *103*, 8577–8593.

Fantini, J., and Barrantes, F. (2013). How cholesterol interacts with membrane proteins: an exploration of cholesterol-binding sites including CRAC, CARC, and tilted domains. Front. Physiol. *4*, 31.

Garcia-Gonzalo, F.R., Phua, S.C., Roberson, E.C., Garcia, G., III, Abedin, M., Schurmans, S., Inoue, T., and Reiter, J.F. (2015). Phosphoinositides regulate ciliary protein trafficking to modulate hedgehog signaling. Dev. Cell *34*, 400–409.

Genheden, S., Essex, J.W., and Lee, A.G. (2017). G protein coupled receptor interactions with cholesterol deep in the membrane. Biochim. Biophys. Acta *1859*, 268–281.

Gimpl, G. (2016). Interaction of G protein coupled receptors and cholesterol. Chem. Phys. Lipids *199*, 61–73.

Gong, X., Qian, H., Cao, P., Zhao, X., Zhou, Q., Lei, J., and Yan, N. (2018). Structural basis for the recognition of sonic hedgehog by human Patched1. Science *361*, eaas8935.

Gordon, R.E., Zhang, L., Peri, S., Kuo, Y.-M., Du, F., Egleston, B.L., Ng, J.M.Y., Andrews, A.J., Astsaturov, I., Curran, T., et al. (2018). Statins synergize with Hedgehog pathway inhibitors for treatment of medulloblastoma. Clin. Cancer Res. *24*, 1375–1388.

Grouleff, J., Irudayam, S.J., Skeby, K.K., and Schiøtt, B. (2015). The influence of cholesterol on membrane protein structure, function, and dynamics studied by molecular dynamics simulations. Biochim. Biophys. Acta *1848*, 1783–1795.

Gu, R.X., Ingolfsson, H.I., de Vries, A.H., Marrink, S.J., and Tieleman, D.P. (2017). Ganglioside-lipid and ganglioside-protein interactions revealed by coarse-grained and atomistic molecular dynamics simulations. J. Phys. Chem. B *121*, 3262–3275.

Gupta, K., Donlan, J.A.C., Hopper, J.T.S., Uzdavinys, P., Landreh, M., Struwe, W.B., Drew, D., Baldwin, A.J., Stansfeld, P.J., and Robinson, C.V. (2017). The role of interfacial lipids in stabilizing membrane protein oligomers. Nature *541*, 421–424.

Gupta, K., Li, J., Liko, I., Gault, J., Bechara, C., Wu, D., Hopper, J.T.S., Giles, K., Benesch, J.L.P., and Robinson, C.V. (2018). Identifying key membrane protein lipid interactions using mass spectrometry. Nat. Protoc. *13*, 1106.

Hansen, S.B., Tao, X., and MacKinnon, R. (2011). Structural basis of PIP_2 activation of the classical inward rectifier K^+ channel Kir2.2. Nature *477*, 495–498.

Hanson, M.A., Cherezov, V., Griffith, M.T., Roth, C.B., Jaakola, V.-P., Chien, E.Y.T., Velasquez, J., Kuhn, P., and Stevens, R.C. (2008). A specific cholesterol binding site is established by the 2.8 Å structure of the human β_2-adrenergic receptor. Structure *16*, 897–905.

Hedger, G., Koldsø, H., and Sansom, M.S.P. (2016a). Free energy landscape of lipid interactions with regulatory binding sites on the transmembrane domain of the EGF receptor. J. Phys. Chem. B *120*, 8154–8163.

Hedger, G., Rouse, S.L., Domański, J., Chavent, H., Koldsø, H., and Sansom, M.S.P. (2016b). Lipid loving ANTs: molecular simulations of cardiolipin interactions and the organization of the adenine nucleotide translocase in model mitochondrial membranes. Biochemistry *55*, 6238–6249.

Hedger, G., and Sansom, M.S.P. (2016). Lipid interaction sites on channels, transporters and receptors: recent insights from molecular dynamics simulations. Biochim. Biophys. Acta *1858*, 2390–2400.

Hedger, G., Sansom, M.S.P., and Koldsø, H. (2015). The juxtamembrane regions of human receptor tyrosine kinases exhibit conserved interaction sites with anionic lipids. Sci. Rep. *5*, 9198.

Hess, B., Bekker, H., Berendsen, H.J.C., and Fraaije, J.G.E.M. (1997). LINCS: a linear constraint solver for molecular simulations. J. Comput. Chem. *18*, 1463–1472.

Hess, B., Kutzner, C., van der Spoel, D., and Lindahl, E. (2008). GROMACS 4: algorithms for highly efficient, load-balanced, and scalable molecular simulation. J. Chem. Theory Comput. *4*, 435–447.

Huang, P., Nedelcu, D., Watanabe, M., Jao, C., Kim, Y., Liu, J., and Salic, A. (2016). Cellular cholesterol directly activates smoothened in Hedgehog signaling. Cell *166*, 1176–1187.

Huang, P., Zheng, S., Wierbowski, B.M., Kim, Y., Nedelcu, D., Aravena, L., Liu, J., Kruse, A.C., and Salic, A. (2018). Structural basis of Smoothened activation in Hedgehog signaling. Cell *174*, 312–324.

Hub, J.S., de Groot, B.L., and van der Spoel, D. (2010). g_wham—a free weighted histogram analysis implementation including robust error and autocorrelation estimates. J. Chem. Theory Comput. *6*, 3713–3720.

Hulce, J.J., Cognetta, A.B., Niphakis, M.J., Tully, S.E., and Cravatt, B.F. (2013). Proteome-wide mapping of cholesterol-interacting proteins in mammalian cells. Nat. Methods *10*, 259.

Humphrey, W., Dalke, A., and Schulten, K. (1996). VMD - visual molecular dynamics. J. Mol. Graph *14*, 33–38.

Ingólfsson, H.I., Melo, M.N., van Eerden, F.J., Arnarez, C., Lopez, C.A., Wassenaar, T.A., Periole, X., de Vries, A.H., Tieleman, D.P., and Marrink, S.J. (2014). Lipid organization of the plasma membrane. J. Am. Chem. Soc. *136*, 14554–14559.

Kalli, A.C., and Reithmeier, R.A.F. (2018). Interaction of the human erythrocyte Band 3 anion exchanger 1 (AE1, SLC4A1) with lipids and glycophorin A: molecular organization of the Wright (Wr) blood group antigen. PLoS Comput. Biol. *14*, e1006284.

Koldsø, H., Shorthouse, D., Hélie, J., and Sansom, M.S.P. (2014). Lipid clustering correlates with membrane curvature as revealed by molecular simulations of complex lipid bilayers. PLoS Comput. Biol. *10*, e1003911.

Laganowsky, A., Reading, E., Allison, T.M., Ulmschneider, M.B., Degiacomi, M.T., Baldwin, A.J., and Robinson, C.V. (2014). Membrane proteins bind lipids selectively to modulate their structure and function. Nature *510*, 172–175.

Lee, A.G. (2018). A database of predicted binding sites for cholesterol on membrane proteins, deep in the membrane. Biophys. J. *115*, 522–532.

Lee, J.Y., and Lyman, E. (2012). Predictions for cholesterol interaction sites on the A2A adenosine receptor. J. Am. Chem. Soc. *134*, 16512–16515.

Li, H., and Papadopoulos, V. (1998). Peripheral-type benzodiazepine receptor function in cholesterol transport. Identification of a putative cholesterol recognition/interaction amino acid sequence and consensus pattern. Endocrinology *139*, 4991–4997.

Liko, I., Degiacomi, M.T., Mohammed, S., Yoshikawa, S., Schmidt, C., and Robinson, C.V. (2016). Dimer interface of bovine cytochrome c oxidase is influenced by local posttranslational modifications and lipid binding. Proc. Natl. Acad. Sci. U S A *113*, 8230–8235.

Liu, S.L., Sheng, R., Jung, J.H., Wang, L., Stec, E., O'Connor, M.J., Song, S., Bikkavilli, R.K., Winn, R.A., Lee, D., et al. (2017). Orthogonal lipid sensors identify transbilayer asymmetry of plasma membrane cholesterol. Nat. Chem. Biol. *13*, 268–274.

Luchetti, G., Sircar, R., Kong, J.H., Nachtergaele, S., Sagner, A., Byrne, E.F.X., Covey, D.F., Siebold, C., and Rohatgi, R. (2016). Cholesterol activates the G-protein coupled receptor smoothened to promote Hedgehog signaling. Elife *5*, e20304.

Marrink, S.J., de Vries, A.H., Harroun, T.A., Katsaras, J., and Wassall, S.R. (2008). Cholesterol shows preference for the interior of polyunsaturated lipid membranes. J. Am. Chem. Soc. *130*, 10–11.

Marrink, S.J., Risselada, J., Yefimov, S., Tieleman, D.P., and de Vries, A.H. (2007). The MARTINI force field: coarse grained model for biomolecular simulations. J. Phys. Chem. B *111*, 7812–7824.

Melo, M.N., Ingólfsson, H.I., and Marrink, S.J. (2015). Parameters for Martini sterols and hopanoids based on a virtual-site description. J. Chem. Phys. *143*, 243152.

Monticelli, L., Kandasamy, S.K., Periole, X., Larson, R.G., Tieleman, D.P., and Marrink, S.J. (2008). The MARTINI coarse grained force field: extension to proteins. J. Chem. Theory Comput. *4*, 819–834.

Myers, B.R., Neahring, L., Zhang, Y., Roberts, K.J., and Beachy, P.A. (2017). Rapid, direct activity assays for Smoothened reveal Hedgehog pathway regulation by membrane cholesterol and extracellular sodium. Proc. Natl. Acad. Sci. U S A *114*, E11141–E11150.

Myers, B.R., Sever, N., Chong, Y.C., Kim, J., Belani, J.D., Rychnovsky, S., Bazan, J.F., and Beachy, P.A. (2013). Hedgehog pathway modulation by multiple lipid binding sites on the Smoothened effector of signal response. Dev. Cell *26*, 346–357.

Nachtergaele, S., Mydock, L.K., Krishnan, K., Rammohan, J., Schlesinger, P.H., Covey, D.F., and Rohatgi, R. (2012). Oxysterols are allosteric activators of the oncoprotein smoothened. Nat. Chem. Biol. *8*, 211.

Nakatsu, F. (2015). A phosphoinositide code for primary cilia. Dev. Cell *34*, 379–380.

Olsson, M.H.M., Sondergaard, C.R., Rostkowski, M., and Jensen, J.H. (2011). PROPKA3: consistent treatment of internal and surface residues in empirical pK(a) predictions. J. Chem. Theory Comput. *7*, 525–537.

Oostenbrink, C., Villa, A., Mark, A.E., and Van Gunsteren, W.F. (2004). A biomolecular force field based on the free enthalpy of hydration and solvation: the GROMOS force-field parameter sets 53A5 and 53A6. J. Comput. Chem. *25*, 1656–1676.

Parrinello, M., and Rahman, A. (1981). Polymorphic transitions in single-crystals - a new molecular-dynamics method. J. Appl. Phys. *52*, 7182–7190.

Patrick, J.W., Boone, C.D., Liu, W., Conover, G.M., Liu, Y., Cong, X., and Laganowsky, A. (2018). Allostery revealed within lipid binding events to membrane proteins. Proc. Natl. Acad. Sci. U S A *115*, 2976–2981.

Periole, X., Cavalli, M., Marrink, S.J., and Ceruso, M.A. (2009). Combining an elastic network with a coarse-grained molecular force field: structure, dynamics, and intermolecular recognition. J. Chem. Theory Comput. *5*, 2531–2543.

Prasanna, X., Chattopadhyay, A., and Sengupta, D. (2014). Cholesterol modulates the dimer interface of the β_2-adrenergic receptor via cholesterol occupancy sites. Biophys. J. *106*, 1290–1300.

Qi, X., Schmiege, P., Coutavas, E., and Li, X. (2018a). Two Patched molecules engage distinct sites on Hedgehog yielding a signaling-competent complex. Science *362*, eaas8843.

Qi, X., Schmiege, P., Coutavas, E., Wang, J., and Li, X. (2018b). Structures of human Patched and its complex with native palmitoylated sonic hedgehog. Nature *560*, 128–132.

Rouviere, E., Arnarez, C., Yang, L., and Lyman, E. (2017). Identification of two new cholesterol interaction sites on the A2A adenosine receptor. Biophys. J. *113*, 2415–2424.

Roux, B. (1995). The calculation of the potential of mean force using computer simulations. Comput. Phys. Commun. *91*, 275–282.

Ruprecht, J.J., Hellawell, A.M., Harding, M., Crichton, P.G., McCoy, A.J., and Kunji, E.R.S. (2014). Structures of yeast mitochondrial ADP/ATP carriers support a domain-based alternating-access transport mechanism. Proc. Natl. Acad. Sci. U S A *111*, E426–E434.

Schmidt, M.R., Stansfeld, P.J., Tucker, S.J., and Sansom, M.S.P. (2013). Simulation-based prediction of phosphatidylinositol 4,5-bisphosphate binding to an ion channel. Biochemistry *52*, 279–281.

Scott, K.A., Bond, P.J., Ivetac, A., Chetwynd, A.P., Khalid, S., and Sansom, M.S.P. (2008). Coarse-grained MD simulations of membrane protein-bilayer self-assembly. Structure *16*, 621–630.

Sengupta, D., and Chattopadhyay, A. (2015). Molecular dynamics simulations of GPCR–cholesterol interaction: an emerging paradigm. Biochim. Biophys. Acta *1848*, 1775–1782.

Shaw, D.E., Deneroff, M.M., Dror, R.O., Kuskin, J.S., Larson, R.H., Salmon, J.K., Young, C., Batson, B., Bowers, K.J., Chao, J.C., et al. (2008). Anton, a special-purpose machine for molecular dynamics simulation. Commun. ACM *51*, 91–97.

Sommer, A., and Lemmon, M.A. (2018). Smoothening out the patches. Science *362*, 26–27.

Sondergaard, C.R., Olsson, M.H.M., Rostkowski, M., and Jensen, J.H. (2011). Improved treatment of ligands and coupling effects in empirical calculation and rationalization of pK(a) values. J. Chem. Theory Comput. *7*, 2284–2295.

Stansfeld, P.J., Hopkinson, R., Ashcroft, F.M., and Sansom, M.S.P. (2009). PIP_2-binding site in Kir channels: definition by multiscale biomolecular simulations. Biochemistry *48*, 10926–10933.

Stansfeld, P.J., and Sansom, M.S.P. (2011). From coarse-grained to atomistic: a serial multi-scale approach to membrane protein simulations. J. Chem. Theory Comput. *7*, 1157–1166.

Tironi, I.G., Sperb, R., Smith, P.E., and Gunsteren, W.F.V. (1995). A generalized reaction field method for molecular dynamics simulations. J. Chem. Phys. *102*, 5451–5459.

van der Spoel, D., van Maaren, P.J., and Berendsen, H.J.C. (1998). A systematic study of water models for molecular simulation: derivation of water models optimized for use with a reaction field. J. Chem. Phys. *108*, 10220–10230.

Van Eerden, F.J., Melo, M.N., Frederix, P.W.J.M., and Marrink, S.J. (2017). Prediction of thylakoid lipid binding sites on photosystem II. Biophys. J. *113*, 2669–2681.

van Meer, G., Voelker, D.R., and Feigenson, G.W. (2008). Membrane lipids: where they are and how they behave. Nat. Rev. Mol. Cell Biol. *9*, 112–124.

Wang, C., Wu, H., Evron, T., Vardy, E., Han, G.W., Huang, X.P., Hufeisen, S.J., Mangano, T.J., Urban, D.J., Katritch, V., et al. (2014). Structural basis for Smoothened receptor modulation and chemoresistance to anticancer drugs. Nat. Commun. *5*, 4355.

Wang, C., Wu, H., Katritch, V., Han, G.W., Huang, X.-P., Liu, W., Siu, F.Y., Roth, B.L., Cherezov, V., and Stevens, R.C. (2013). Structure of the human smoothened receptor bound to an antitumour agent. Nature *497*, 338–343.

Weierstall, U., James, D., Wang, C., White, T.A., Wang, D., Liu, W., Spence, J.C.H., Bruce Doak, R., Nelson, G., Fromme, P., et al. (2014). Lipidic cubic phase injector facilitates membrane protein serial femtosecond crystallography. Nat. Commun. *5*, 3309.

Wu, F., Zhang, Y., Sun, B., McMahon, A.P., and Wang, Y. (2017). Hedgehog signaling: from basic biology to cancer therapy. Cell Chem. Biol. *24*, 252–280.

Yeagle, P.L. (2014). Non-covalent binding of membrane lipids to membrane proteins. Biochim. Biophys. Acta *1838*, 1548–1559.

Yen, H.-Y., Hoi, K.K., Liko, I., Hedger, G., Horrell, M.R., Song, W., Wu, D., Heine, P., Warne, T., Lee, Y., et al. (2018). PtdIns(4,5)P2 stabilizes active states of GPCRs and enhances selectivity of G-protein coupling. Nature *559*, 423–427.

Zeppelin, T., Ladefoged, L.K., Sinning, S., Periole, X., and Schiøtt, B. (2018). A direct interaction of cholesterol with the dopamine transporter prevents its out-to-inward transition. PLoS Comput. Biol. *14*, e1005907.

Zhang, X., Zhao, F., Wu, Y., Yang, J., Han, G.W., Zhao, S., Ishchenko, A., Ye, L., Lin, X., Ding, K., et al. (2017). Crystal structure of a multi-domain human smoothened receptor in complex with a super stabilizing ligand. Nat. Commun. *8*, 15383.

Zhang, Y., Bulkley, D.P., Roberts, K.J., Xin, Y., Asarnow, D., Sharma, A., Myers, B.R., Cho, W., Cheng, Y., and Beachy, P.A. (2018). Structural basis for cholesterol transport-like activity of the hedgehog receptor patched. Cell *175*, 1352–1364.

Zocher, M., Zhang, C., Rasmussen, S.G.F., Kobilka, B.K., and Muller, D.J. (2012). Cholesterol increases kinetic, energetic, and mechanical stability of the human beta(2)-adrenergic receptor. Proc. Natl. Acad. Sci. U S A *109*, E3463–E3472.

STAR★METHODS

KEY RESOURCES TABLE

REAGENT or RESOURCE	SOURCE	IDENTIFIER
Software and Algorithms		
Gromacs 4.6	(Hess et al., 2008)	www.gromacs.org
Martini force field 2	(de Jong et al., 2013)	www.cgmartini.nl
GROMOS53a6 force field	(Oostenbrink et al., 2004)	www.gromacs.org/Downloads/User_contributions/Force_fields
VMD 1.9.2	(Humphrey et al., 1996)	www.ks.uiuc.edu/Research/vmd
SMO human structure	PDB: 5L7D	www.rcsb.org
SMO *X. laevis* structure	PDB: 6D32	www.rcsb.org

CONTACT FOR REAGENT AND RESOURCE SHARING

Further information and requests for resources and reagents should be directed to and will be fulfilled by the Lead Contact Mark Sansom (mark.sansom@bioch.ox.ac.uk).

METHOD DETAILS

SMO Model Building

The SMO model used in simulations was based on the near full-length structure (PDB entry 5L7D) (Byrne et al., 2016). The primary goal of these simulations was to characterize cholesterol interactions with the transmembrane domain. As such the structure was truncated at position 191, yielding a construct (residues 192-549) consisting of the LD and 7TMD. This has previously been shown to be a stable unit for which multiple structures exist (Wang et al., 2013, 2014; Weierstall et al., 2014), and functionally viable in a membrane environment (Myers et al., 2017). Simulating only the LD and 7TMD construct enabled us to create smaller simulation boxes and expedite data collection. Side chain ionization states were modelled using pdb2gmx (Histidine) and PropKa (All other residues) (Olsson et al., 2011; Sondergaard et al., 2011). The N and C-termini were treated as neutral. The stabilizing and inactivating V329F mutation was left untouched. Intracellular loop 3 (occupied by the BRIL fusion in the 5L7D crystal structure) was modelled using coordinates from the PDB entry 4N4W (Wang et al., 2014). The protein structure was then energy minimized using the steepest descent algorithm implemented in GROMACS (Hess et al., 2008).

Coarse-Grained Simulations

The minimized protein structure was converted to a CG representation using the Martini 2.2 force field (de Jong et al., 2013; Monticelli et al., 2008). Tertiary structure was modelled using an Elnedyn network with a cutoff distance of 0.9 nm and a force constant of 500 kJ/mol/nm^2. This approach prevents significant conformational deviations from the initial reference coordinates, while preserving local dynamics (Periole et al., 2009). The CG protein was centered in a simulation box of dimensions 10 x 10 x 13 nm, containing 280 randomly oriented 1-palmitoyl-2-oleoyl-sn-glycero-3-phosphocholine (POPC) lipids. The system was solvated using the standard Martini water model (Marrink et al., 2007), and neutralized with 0.15 M NaCl, before being subjected to 100 ns of CG simulation to permit the self-assembly of a lipid bilayer. This approach allows the protein to dynamically adopt its optimum orientation within the bilayer (Scott et al., 2008). Randomly selected POPC lipids were subsequently exchanged (Koldsø et al., 2014) for cholesterol molecules, to create mixed membranes of specified lipid composition (Table 1). Exchanges were only allowed outside a 2.5 nm cutoff distance from the protein surface, to avoid potential bias arising from fortuitous pre-placement. This process was repeated for each individual repeat simulation, so as to create different random initial lipid configurations. An analogous process was performed for the 5-component plasma membrane mimetic simulations. Lipid compositions were chosen based on experimental lipidomics (van Meer et al., 2008). The standard Martini cholesterol parameters correspond to those of (Marrink et al., 2008), whilst the virtual site Martini cholesterol parameters were taken from (Melo et al., 2015). PIP_2 parameters were created in-house as previously described (Stansfeld et al., 2009).

Temperature and semi-isotropic pressure were controlled at 310 K and 1 bar using the Berendsen barostat and Berendsen thermostat, with a coupling constants of 4 ps (Berendsen et al., 1984). Van der Waals interactions were smoothly shifted off between 0.9 nm and 1.2 nm. Modelling of electrostatics utilized the reaction field approach (Tironi et al., 1995), with a Coulomb cutoff of 1.2 nm and a potential shift modifier. Equations of motion were integrated with a 20 fs timestep, using the leapfrog algorithm implemented in GROMACS. Covalent bonds were constrained to their equilibrium values using the LINCS algorithm (Hess et al., 1997).

Analysis

Simulation data was analysed using VMD (Humphrey et al., 1996), tools implemented in GROMACS (Hess et al., 2008), and in-house protocols.

Contact Analysis

Contact analysis was performed using in-house protocols (Koldsø et al., 2014). A ‘contact’ was counted if any particle of a cholesterol/PIP_2 molecule came within the cutoff distance of any particle of a given residue. Further this counting was not capped at 1. Thus if three particles of a cholesterol molecule were simultaneously contacting a given residue (a rare occurrence) then 3 contacts would be counted for that residue. (This was done because we consider that 3 contacts counted reveal a stronger interaction than just one contact.) Per residue contacts were computed in this manner for each frame of the simulation, and the mean calculated across all frames. Protein-lipid contact analysis employed a cutoff distance of 0.6 nm, based on radial distribution functions for CG lipid molecules (Hedger et al., 2015). Likewise, annular lipids were calculated as those within 0.6 nm of the protein surface.

Density Analysis

2D density maps were computed using locally developed python tools (Kalli and Reithmeier, 2018). Density calculations were performed by drawing a grid over the simulation box, and counting how many cholesterol particles occupied each unit of the grid for each frame of the simulation. Thus, as for contacts, density maps (see above) were based on particles within cholesterol rather than a single centre-of-mass point for each cholesterol. We confirmed (data not shown) that this approach did not alter the essential features of the resultant 2D density maps. The 2D density maps shown correspond to the mean across all frames, normalised for the user’s choice of grid size by dividing by the area of each grid unit. Rotation and translation of the protein were alleviated by performing the calculation on a trajectory which had been fitted to the protein backbone using trjconv -fit option in GROMACS.

Potential of Mean Force Calculations

Potential of mean force (PMF) calculations were performed using a protocol previously described (Hedger et al., 2016a, 2016b). All 7TMD PMF calculations were conducted in a PC only bilayer with a single cholesterol molecule initially bound to the SMO TMD. This simple system was chosen to accelerate convergence during the PMF simulations (Domański et al., 2017). A one-dimensional reaction coordinate was generated using a steered molecular dynamics (SMD) simulation to pull the cholesterol from the bound to unbound state. Each cholesterol molecule was pulled away from the protein over a distance of 3.5 nm along a coordinate orthogonal to the protein surface. The SMD was performed at a rate of 0.1 nm/ns (F_c = 1000 kJ/mol/nm^2) via application of a force to the ROH particle of cholesterol. Within 7TMD PMF calculations, position restraints (F_c = 400 kJ/mol/nm^2) were also applied in the *X*–*Y* plane to the backbone particles of V240, P369, and L464. These residues are distal from the respective cholesterol binding sites. In addition, weaker positional restraints (F_c = 50 kJ/mol/nm^2) were applied to the ROH particle of cholesterol in the *Y* direction. Application of such restraints acted to prevent rotation of the protein, and translational ‘‘following’’ of cholesterol molecules as they were pulled away. Umbrella sampling simulations employed a window separation of 0.1 nm, using initial conformations extracted from the SMD simulation. Each window was run for 1 μs, with umbrella biasing potentials (F_c = 1000 kJ/mol/nm^2) applied between the center of mass of the triad of restrained residues and the ROH particle of cholesterol. The subject lipid was treated separately from bulk lipids for temperature and pressure coupling. Approximately 32 umbrella sampling simulations were run per PMF. PMF profiles were constructed using the GROMACS implementation (g_wham) of the weighted histogram analysis method (WHAM) (Hub et al., 2010). Bayesian bootstrapping with 200 bootstraps was used to estimate the errors for each profile. Convergence was assessed by comparing profiles calculated from independent 100 ns segments of simulation time (Figure S5B).

Atomistic Simulations

Atomistic simulations were run using the GROMOS53a6 force field (Oostenbrink et al., 2004). The system was solvated using the SPC water model (van der Spoel et al., 1998) and neutralized with NaCl to a concentration of 0.15 M. Periodic boundary conditions were applied, with a simulation time step of 2 femtoseconds. A V-rescale thermostat (Bussi et al., 2007) was used to maintain temperature around 310 K, with a coupling constant of 0.1 ps, whilst pressure was controlled at 1 bar through coupling to a Parrinello-Rahman barostat (Parrinello and Rahman, 1981), with a coupling constant of 1 ps. Particle Mesh Ewald (PME) (Essmann et al., 1995) was applied to model long-range electrostatics. Van der Waals interactions were cut off at 1.2 nm. The LINCS algorithm was used to constrain covalent bond lengths (Hess et al., 1997).

DATA AND SOFTWARE AVAILABILITY

Coordinates of the model generated by this study (as representative frames from atomistic simulations revealing the interactions of the SMO transmembrane domain with cholesterol) are available from the lead contact.

Structure, Volume *27*

Supplemental Information

Cholesterol Interaction Sites on the Transmembrane Domain of the Hedgehog Signal Transducer and Class F G Protein-Coupled Receptor Smoothened

George Hedger, Heidi Koldsø, Matthieu Chavent, Christian Siebold, Rajat Rohatgi, and Mark S.P. Sansom

Supplemental Information

Cholesterol and PIP_2 interaction sites on the transmembrane domain of the hedgehog signal transducer and Class F GPCR Smoothened

George Hedger, Heidi Koldsø, Matthieu Chavent, Christian Siebold, Rajat Rohatgi, and Mark S. P. Sansom

Table S1. Comparison of lateral lipid-protein binding energies for integral membrane proteins extracted from CG PMF calculations *via* umbrella sampling. Related to Figure 6.

Protein	Lipid	Well-depth (kJ/mol)	Membrane composition
Cytochrome C Oxidase[a] (Arnarez et al., 2013; Arnarez et al., 2016)	Cardiolipin^{-2}	-32 (Site I, II), -9 (Site III)	PC (100%), also tested in PC(50%):PE(50%) for Site II yielding no significant deviation.
	Cardiolipin^{0}	-21 (Site II)	PC (100%)
	PG^{-1}, PG^{-2}	-19, -24 (Site II)	
	PC	-10 (Site II)	PC (100%)
	TGL	< RT (Site II)	PC (100%)
ADP/ATP Translocase (Hedger et al., 2016b)	Cardiolipin^{-2}	-22, -20, -24 (Site I, II, III)	PC (100%)
	PS	-17 (Site I)	PC (100%)
	PC	< RT	PC (100%)
Epidermal Growth Factor Receptor Transmembrane Dimer (Hedger et al., 2016a)	PIP_2^{-5}, PIP_2^{-3}, PIP_2^{c}	-42, -35, -13	PC (100%)
	PS	-9	PC (100%)
	GM3, GM3 (-Neu5Ac)	-9, -4	PC (100%)
	PG	-5	PC (100%)
	PC	< RT	PC (100%)
Kir2.2[b] (Domański et al., 2017)	PIP_2^{-5}	-45	PC (100%)
P-glycoprotein (Domicevica et al., 2018)	Cholesterol	-25, -6, -4 (Sites I, II, III)	PC (100%)
Aquaporin-1[d] (Gu et al., 2017)	GM3	-24 to -19	PC (100%)

[a]These studies include a number of other calculations for various tail saturation patterns and cardiolipin derivatives. Only the major lipid types are catalogued here. See the original references for further details (Arnarez et al., 2013; Arnarez et al., 2016).[b]Calculations for this system were performed using replica exchange umbrella sampling. [c,d]GM3 profiles for interaction with Aquaporin-1 were performed using a several water models, only the range of values obtained is given here. See the original paper (Gu et al., 2017) for further details.

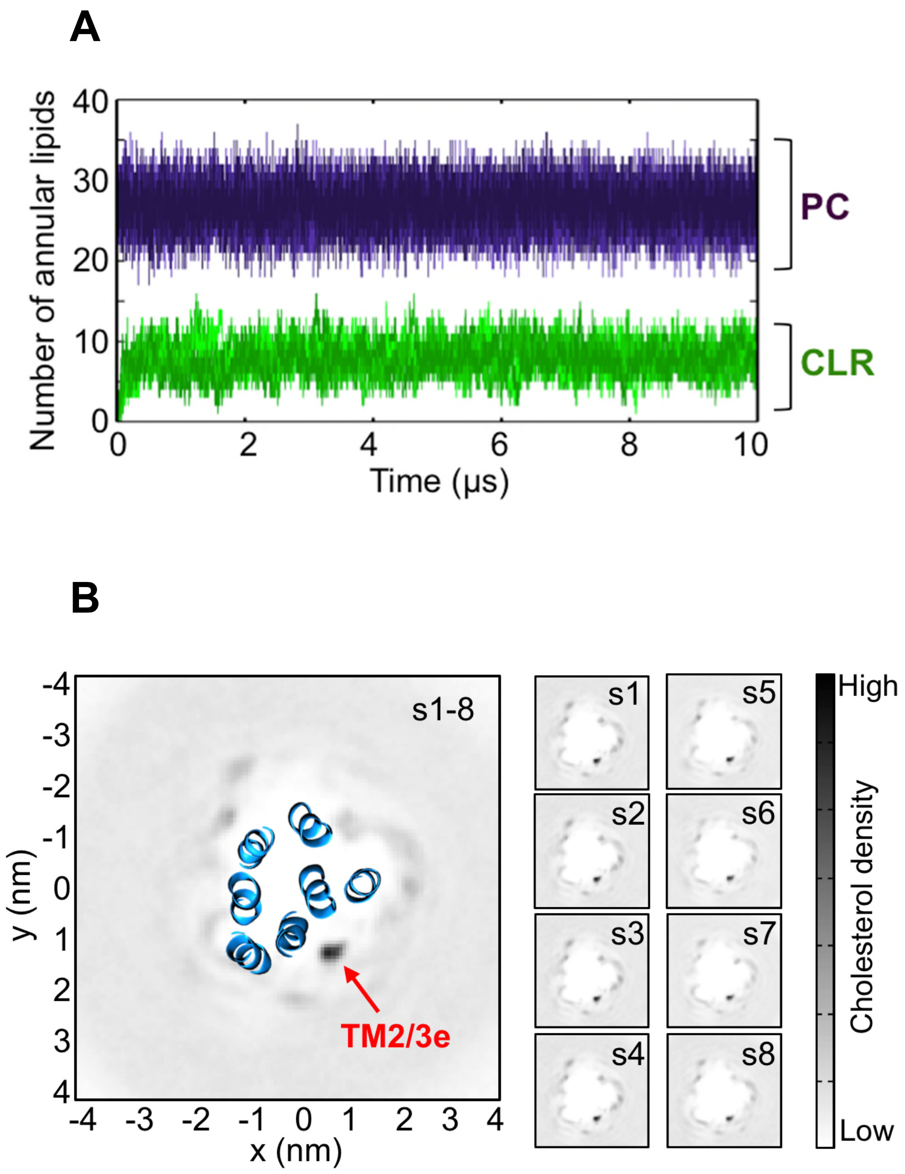

Figure S1: Convergence tests. Related to Figure 2.

A. Annular lipid composition. Number of PC and cholesterol (CLR) annular are shown as a function of simulation time. Data is shown for 8 x 10 µs of CGMD. Annular lipids are defined as those within an 6 Å cutoff distance from the protein. **B.** Convergence of independent CG-MD simulations. 2D time-averaged density projections for membrane cholesterol around SMO. Left: Density averaged over 8 independent simulations each of 10 µs duration of CGMD, Right: Density projections from each individual repeat simulation. Each repeat simulation (s1-8) was initiated from a different random distribution of lipids, waters, and ions around the protein. Invariably all simulations converged on the same density pattern, pointing to TM2/3e as a putative cholesterol interaction site.

A

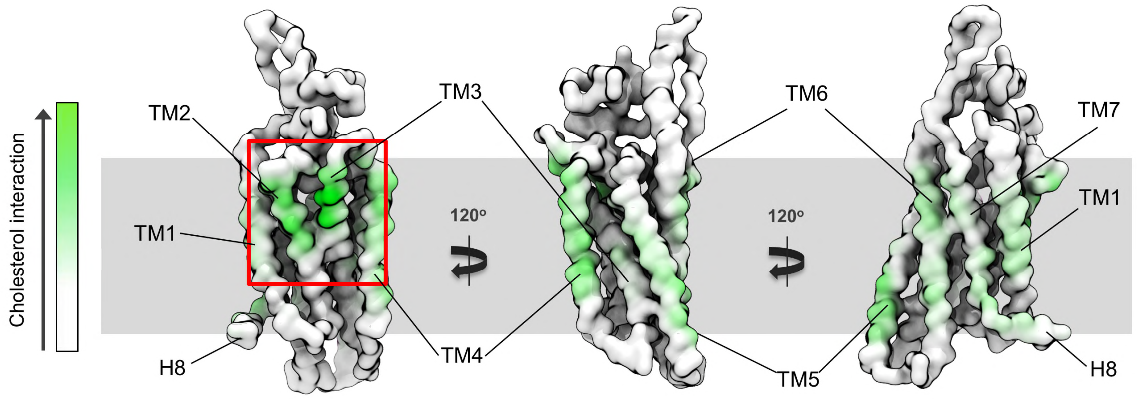

B

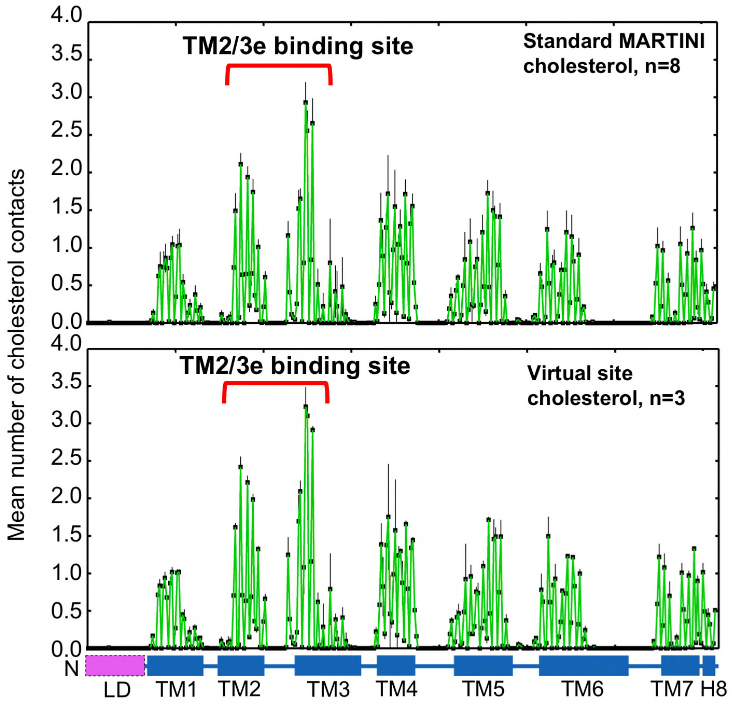

Figure S2. Cholesterol contacts. Related to Figure 3.

A. Global view of cholesterol contacts mapped onto structure. Each residue is colored according to the mean number of cholesterol contacts formed per frame of the simulation ("cholesterol interaction"), from white (no contact) to green (high contact) using contacts extracted from 8 x 10 µs of CGMD. The TM2/3e binding site is delineated by a red box. Contact analysis was performed using a 6 Å distance cutoff to define 'contact', based on the radial distribution function for CG Martini lipid-protein interactions. Further details of the contact analysis are described in the Methods section. **B.** Comparing cholesterol parameters. Cholesterol contact maps for (upper panel) the standard cholesterol model (Marrink et al., 2008), and (lower panel) the virtual site cholesterol model (Melo et al., 2015). *n* denotes the number of simulation repeats. Black error bars denote the standard deviation.

A

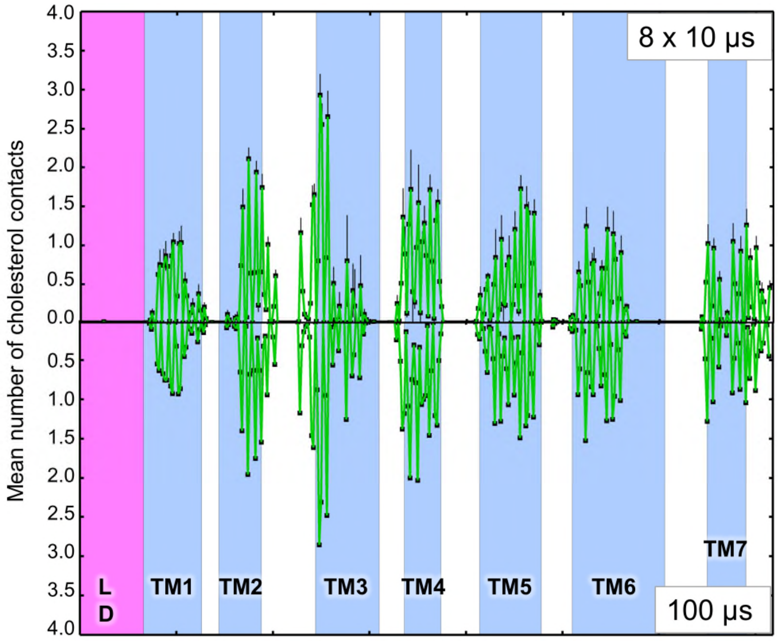

B

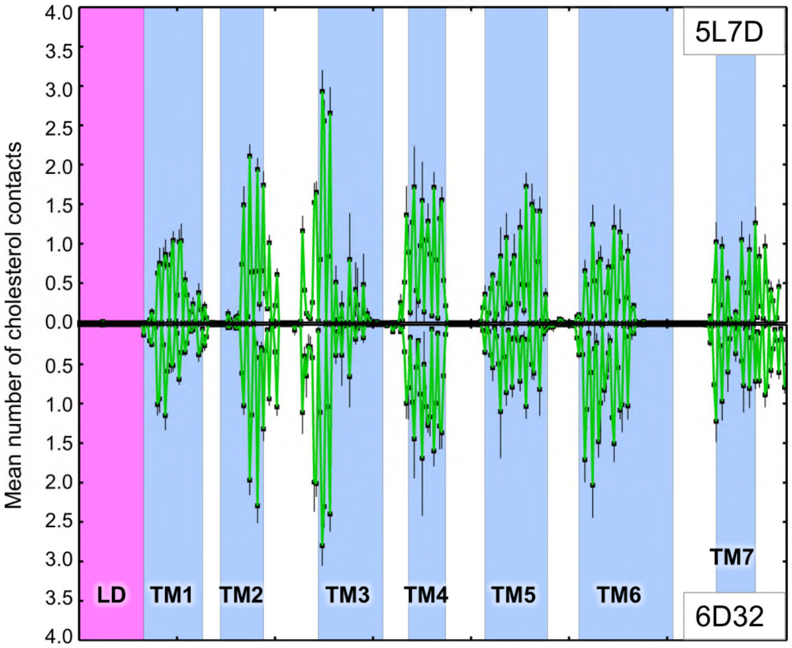

Figure S3. Cholesterol contacts. Related to Figure 3.

A. Time dependent convergence of cholesterol contacts within equilibrium CGMD simulations. Cholesterol contacts are shown for an ensemble of 8 x 10 μs of CGMD (top), and for a single 100 μs CGMD simulation (bottom). Black error bars denote the standard deviation (*n*=8). The contact maps are identical within the errors derived from the smaller ensemble. **B.** Conformational dependence of cholesterol contacts. Cholesterol contacts are shown for a structure of the human 7TMD (PDB id: 5L7D) (top), and for a recently emerged alternative conformational from *X. laevis* (PDB id: 6D32) (bottom). In both cases contacts were extracted from an ensemble of 8 x 10 μs of CGMD. Black error bars denote the standard deviation (*n*=8). The high levels of contact around TM2/3e are preserved in both structures.

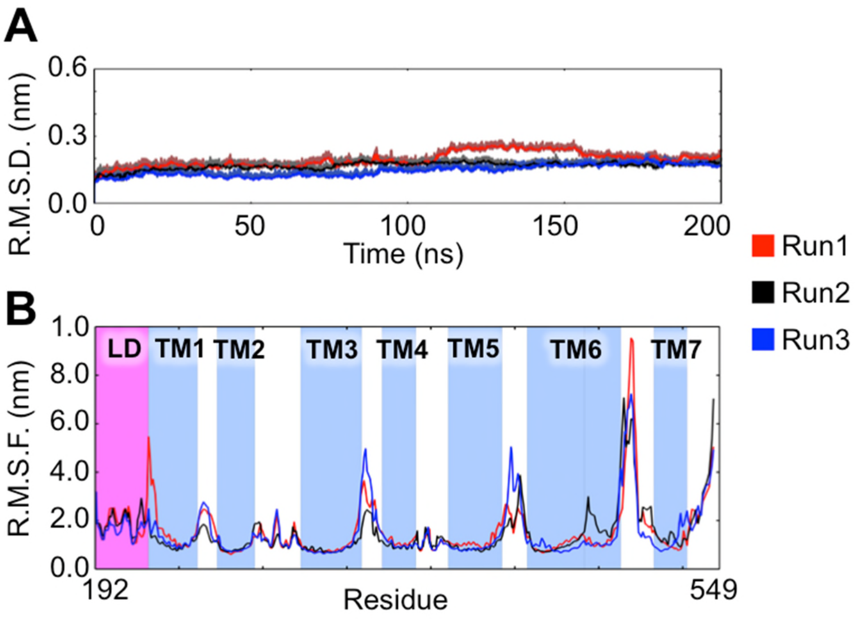

Figure S4. SMO stability within atomistic simulations. Related to Figure 5.

A. Root mean square deviation (RMSD) for Cα particles within helical secondary structure, calculated after fitting to the crystal structure coordinates (Byrne et al., 2016). The values reach a stable plateau of ca. 0.2 nm for all three repeats simulations. **B.** The predominant structural flexibility was seen in disordered loop regions, the C-terminal tail, and particularly ECL3 connecting TM6 and TM7. These observations are in agreement with our previous all-atom simulations of the full-length construct containing the CRD (Byrne et al., 2016).

A

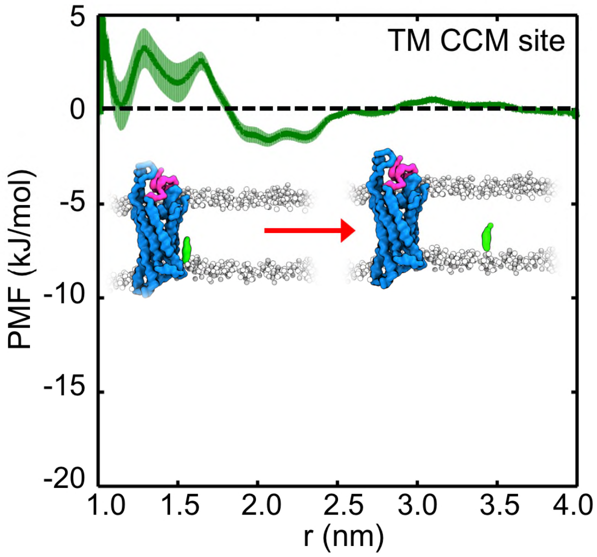

B

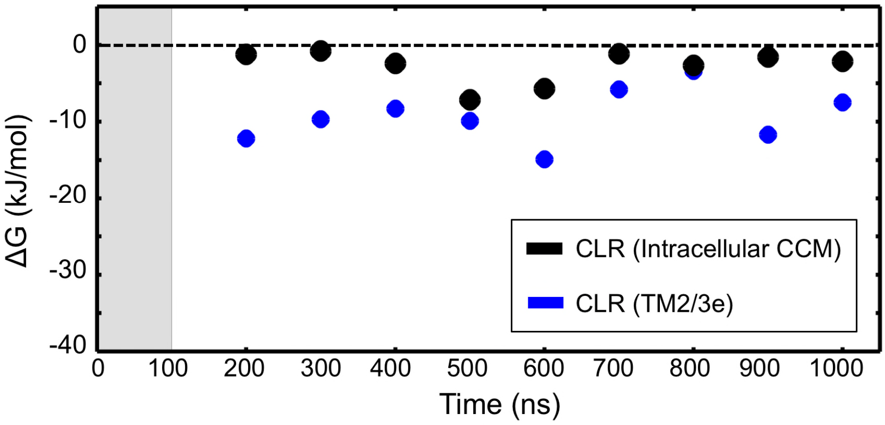

Figure S5. PMF analysis. Related to Figure 6.

A. PMF for cholesterol interaction with cholesterol consensus motif (CCM) (Hanson et al., 2008). Insets depict simulation snapshots of bound and unbound cholesterol. The shaded areas behind each curve indicate the standard deviation estimated from bootstrapping. The well-depth is < 2.5 kJ/mol (RT), indicating no significant interaction at this region, in agreement with the blind predictions of the equilibrium simulations. **B.** Well-depths calculated for multiple, non-overlapping 100 ns segments of CGMD are shown as a function of time. The first 100 ns of each window were excluded as equilibration. The patterns obtained here may be compared to those of Cardiolipin (also shown here) binding to the ADP/ATP Translocase we previously reported (Hedger et al., 2016b).

A

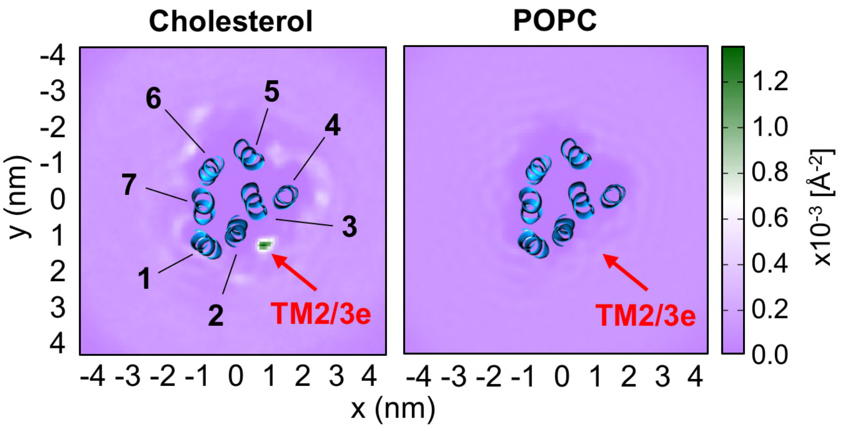

B

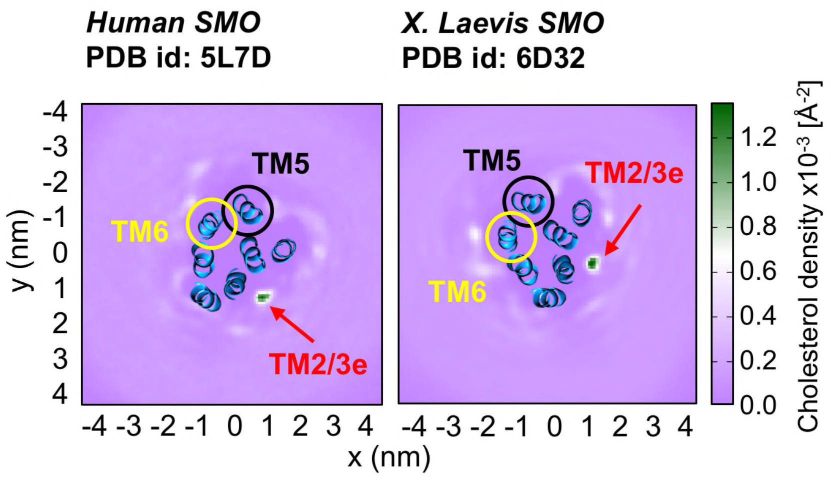

Figure S6: Cholesterol density maps. Related to Figure 2.

A. (left panel) Cholesterol density around SMO computed from 8 x 10 µs of CGMD. (right panel) POPC density around SMO in a set of control CGMD simulations totaling 3 x 10 µs in a POPC bilayer containing no cholesterol. In the absence of cholesterol POPC showed no specific interaction sites, instead forming a characteristic anulus around the protein and interacting in a non-specific fashion. See Methods for details of how density calculations were performed. **B.** Dependence of cholesterol density maps on structure. 2D cholesterol density maps are shown for CGMD simulations using the structure of human SMO (left) and the recently determined alternative conformation of the *X. laevis* structure (right). To aid comparison TM5 is encircled in black and TM6 in yellow. The location of TM2/3e is indicated by a red arrow. The primary cholesterol binding site in both sets of simulations is TM2/3e, with only a low level of diffuse density observed around TM5 and TM6. See Table 1 for a summary of the simulations, and Methods for a description of how the density calculations were performed.

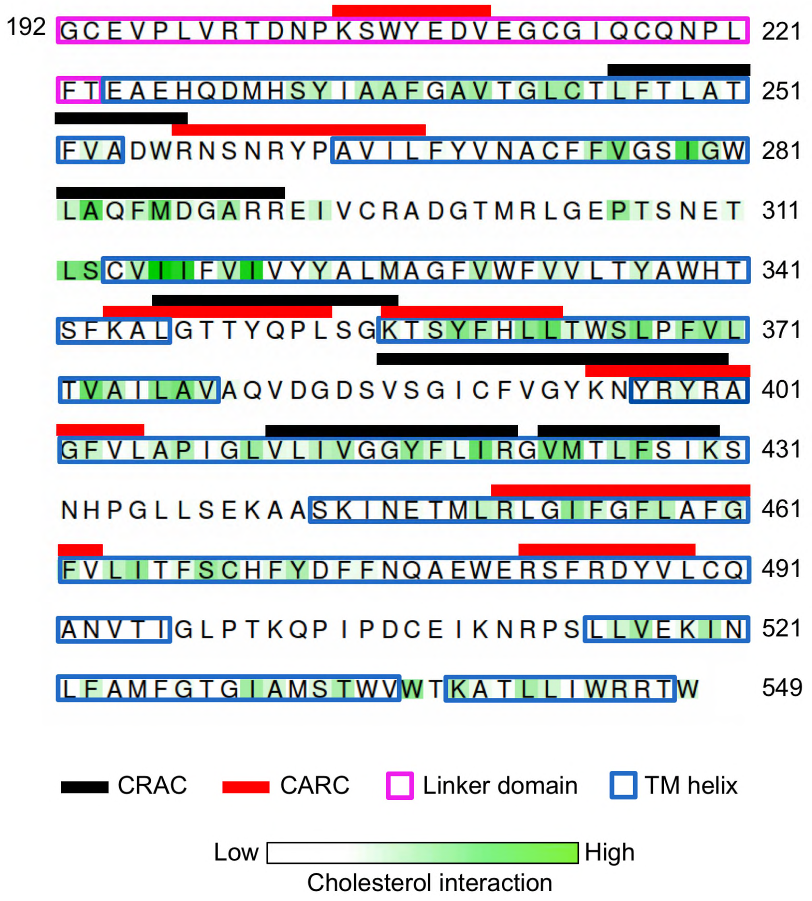

Figure S7. Co-localization of cholesterol contacts with putative cholesterol interaction motifs. Related to Figure 3.

The primary sequence of the simulated construct is shown, colored according to cholesterol interaction, from white (no contact) to green (high contact) using ALINE (Bond and Schuttelkopf, 2009). Contact data was extracted from a 100 µs CGMD simulation. The positions of the linker domain (magenta boxes) and transmembrane helices (blue boxes) are shown according to the DSSP predictions used to assign secondary structure in the simulations. The position of CRAC and CARC motifs are denoted by black and red bars, respectively.
